# A systematic review of empirical and simulation studies evaluating the health impact of transportation interventions

**DOI:** 10.1016/j.envres.2020.109519

**Published:** 2020-07

**Authors:** Ivana Stankov, Leandro M.T. Garcia, Maria Antonietta Mascolli, Felipe Montes, José D. Meisel, Nelson Gouveia, Olga L. Sarmiento, Daniel A. Rodriguez, Ross A. Hammond, Waleska Teixeira Caiaffa, Ana V. Diez Roux

**Affiliations:** a Urban Health Collaborative, Dornsife School of Public Health, Drexel University, 3600 Market St, 7th Floor, Philadelphia, PA, 19104, USA; b UKCRC Centre for Diet and Activity Research (CEDAR), MRC Epidemiology Unit, University of Cambridge School of Clinical Medicine, Cambridge, UK; c Department of Preventive Medicine, University of São Paulo Medical School, São Paulo, Brazil; d Department of Industrial Engineering, Social and Health Complexity Center, Universidad de Los Andes, Bogotá, Colombia; e School of Medicine, Universidad de Los Andes, Cra 1 # 18a-10, Bogotá, Colombia; f Facultad de Ingeniería, Universidad de Ibagué, Carrera 22 Calle 67, Ibagué, 730001, Colombia; g University of California, Berkeley, USA; Department of City and Regional Planning and Institute for Transportation Studies, University of California, Berkeley, USA; h Center on Social Dynamics and Policy, The Brookings Institution, 1775 Massachusetts Ave NW, Washington, DC, 20036, USA; i Brown School at Washington University in St. Louis, One Brookings Drive, St Louis, MO, 36130, USA; j Observatory for Urban Health in Belo Horizonte, School of Medicine, Federal University of Minas Gerais, Belo Horizonte, Brazil

**Keywords:** Transportation, Health, Systematic review, Natural experiment, Complex systems

## Abstract

Urban transportation is an important determinant of health and environmental outcomes, and therefore essential to achieving the United Nation's Sustainable Development Goals. To better understand the health impacts of transportation initiatives, we conducted a systematic review of longitudinal health evaluations involving: a) bus rapid transit (BRT); b) bicycle lanes; c) Open Streets programs; and d) aerial trams/cable cars. We also synthesized systems-based simulation studies of the health-related consequences of walking, bicycling, aerial tram, bus and BRT use.

Two reviewers screened 3302 unique titles and abstracts identified through a systematic search of MEDLINE (Ovid), Scopus, TRID and LILACS databases. We included 39 studies: 29 longitudinal evaluations and 10 simulation studies. Five studies focused on low- and middle-income contexts. Of the 29 evaluation studies, 19 focused on single component bicycle lane interventions; the rest evaluated multi-component interventions involving: bicycle lanes (n = 5), aerial trams (n = 1), and combined bicycle lane/BRT systems (n = 4). Bicycle lanes and BRT systems appeared effective at increasing bicycle and BRT mode share, active transport duration, and number of trips using these modes. Of the 10 simulation studies, there were 9 agent-based models and one system dynamics model. Five studies focused on bus/BRT expansions and incentives, three on interventions for active travel, and the rest investigated combinations of public transport and active travel policies. Synergistic effects were observed when multiple policies were implemented, with several studies showing that sizable interventions are required to significantly shift travel mode choices.

Our review indicates that bicycle lanes and BRT systems represent promising initiatives for promoting population health. There is also evidence to suggest that synergistic effects might be achieved through the combined implementation of multiple transportation policies. However, more rigorous evaluation and simulation studies focusing on low- and middle-income countries, aerial trams and Open Streets programs, and a more diverse set of health and health equity outcomes is required.

## Highlights

• Bike lanes and bus rapid transit systems promote physical activity and active travel.
• Multi-component interventions can act synergistically to increase active travel.
• Few longitudinal health evaluations of aerial trams and open streets programs exist.
• Low-and-middle income countries are understudied in the literature.

## Graphical abstract

This figure represents a word network created by extracting keywords from the paper abstracts included in our systematic review. Each keyword represents a node in the network; its size is proportional to the number of abstracts in which it appears. Keywords are connected if they are found in the same abstract. The colours represent different communities of words as identified using the Louvain method.

**Figure:**
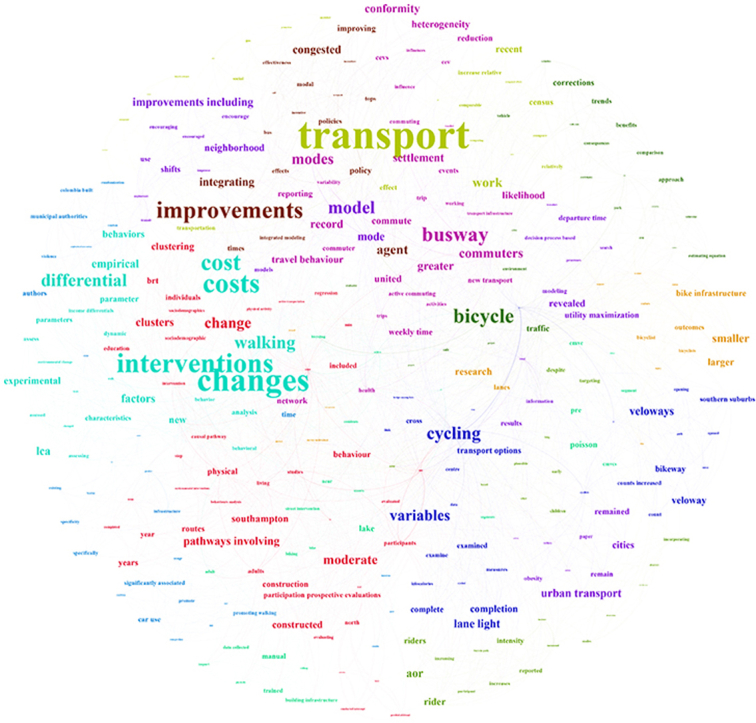

## 1 Introduction

Over the course of the last two centuries, the proportion of the world's population residing in cities has increased more than 10 fold, with more than half of all people living in urban areas (United Nations, 2014). According to the United Nations (United Nations, 2018), especially rapid rates of urbanization are projected to occur in low- and middle-income countries. While the dense intersection of social, natural, and built environments in cities affords numerous health-related benefits, it can also pose serious risks to human health, well-being, and environmental sustainability (Vlahov et al., 2005; Rydin et al., 2012).

Transportation is well recognised as an important feature of urban life and a significant determinant of health and well-being. Beyond the direct associations between transport and traffic accident-related injuries (Litman, 2013), transportation may also influence health and well-being via indirect pathways. For instance, transportation serves an important function in facilitating social interaction and access to a wide range of health-related opportunities, including health care services, employment and educational opportunities (Litman, 2013). The relationships between transportation and health may also transpire through mediating factors such as physical inactivity, and air and noise pollution arising from an overreliance on motorized forms of transportation (Brunekreef and Holgate, 2002; Babisch, 2006; Lee et al., 2012). The design of transportation systems, through their capacity to enable or constrain mobility, can also impact a city's economic growth and urban structure (Becerra et al., 2013). As a determinant of health, transport advantage or disadvantage, including inadequate access to transportation infrastructure and services, can exacerbate social segregation (Lucas, 2012) and impact health inequalities (Borrell et al., 2013).

The importance of transportation is reflected within the Transformative Commitments of the New Urban Agenda (United Nations, 2017) and the United Nation's Agenda for Sustainable Development (UN General Assembly, 2015), which features transportation as essential to achieving the 17 Sustainable Development Goals (United Nations, 2016). Recognising this call to action, international agencies and cities worldwide have expressed a strong interest in the design and implementation of transportation-based policies and initiatives capable of addressing some of the unique challenges faced by rapidly urbanising cities. Four emerging and innovative policies in urban mobility, that have attracted attention and been implemented in both high and low- and middle-income countries (HIC and LMIC, respectively) include: 1) bus rapid transit (or BRT) in over 160 cities (for example, the Mexico City Metrobús, the Lagos BRT in Nigeria and the Spurbus in Germany) (Becerra et al., 2013, BRT+ Centre of Excellence and EMBARQ, 2019); 2) bicycle paths (for example, Santiago's Mapocho Pedaleable in Chile and Denmark's well-known network of paths and bicycle lanes) (Pucher et al., 2010; Becerra et al., 2013); 3) Open Streets programs, which involve the temporary closure of main streets to motorized traffic in order to encourage cycling and other modes of active transport (Kuhlberg et al., 2014; Sarmiento et al., 2017); and 4) aerial trams (i.e, cable cars) designed to connect peripheral hillside neighborhoods or islands with downtown activity nodes (examples include: the Medellín Metrocable in Colombia, the Cable of Constantine in Algeria, and the Roosevelt Island Tramway in New York City) (Alshalalfah et al., 2012).

Despite the growing interest in the health-related impacts of transportation policies, there have been few attempts to evaluate these initiatives. From a public health vantage, it is important to understand what, if any, impact these transportation policies have had on population health and health inequities in both HIC and LMIC. By identifying initiatives that have demonstrated positive public health effects, as well as highlighting gaps in understanding, the synthesis of local and international research can inform decision-making and policy design in cities all over the world.

In addition to health impact evaluations, research employing system-based simulation methods such as agent-based modelling (ABM) and system dynamics (SD) can also be used to elucidate the potential health-related impacts of diverse policies. Large-scale transportation interventions can affect travel behavior, population health, and health inequalities in multiple ways that are hard to anticipate due to the complex and dynamic relations between these policies and the system on which they act. System-based simulation methods are able to capture complex mechanisms not readily accommodated by other analytical approaches, including non-linearity, feedback loops, individual and collective adaptation to changes in environmental and social contexts, self-organization, and emergence (Jayasinghe, 2011). Simulation models are often used to assess the health-related impacts of large-scale, complex population-level interventions that are often prohibitively expensive or impractical to trial in the real world (Hammond, 2015). For example, elucidating the dynamic mechanisms through which transportation policies impact population health can improve the design of more effective initiatives and reduce the potential for undesirable or unintended consequences. Another advantage of simulation modelling is its capacity to facilitate policy prioritization and planning by affording a platform through which the differential or combined health impact of diverse policies may be compared (Hammond, 2015). While there have been a number of systematic reviews of simulation-based methods applied to the study of non-communicable diseases (Nianogo and Arah, 2015; Li et al., 2016), there has been no attempt to review models simulating the impact of transportation mode choices and transport-focused initiatives on health outcomes, more broadly.

This systematic review has two broad aims. The first is to summarize existing evidence (longitudinal empirical and simulation-based) on the influence of four innovative transportation policies, programs, and investments (i.e., BRT, bicycle lanes, Open Streets programs, and aerial trams) on health-related behavior and or health outcomes. And second, based on the synthesized evidence, this review will outline key recommendations for future research.

## 2 Methods

### 2.1 Study design

A systematic review of the peer-reviewed literature was conducted with a focus on identifying: 1) primary studies evaluating the influence of BRT, bicycle lanes, Open Streets programs, and aerial trams on health-related behavior and or health outcomes; and 2) system-based simulation studies exploring the links between transportation mode choice, BRT, bicycle lanes, Open Streets programs, and aerial tram policies, and health-related behavior and or health outcomes. The PRISMA checklist was used to ensure methodological rigor (Moher et al., 2009) and the systematic review was registered with the International Prospective Register of Systematic Reviews (PROSPERO No. CRD42018093172). A narrative synthesis was conducted to characterize studies and investigate the health impacts of the four types of applied and simulated policies and projects.

### 2.2 Search strategy

A search strategy seeking to identify all relevant reviews and primary studies was developed by IS and LMTG, and further refined through consultation with a project working group. Four electronic databases formed the focus of the literature search: MEDLINE (Ovid), Scopus, Transportation Research International Documentation (TRID) and LILACS. These databases were selected because of their coverage of literature from both HIC and LMIC. Using a combination of keywords, MeSH terms, and phrases, a relatively broad search strategy was employed to ensure all relevant studies published from the year 2000 and onwards were identified. The basic search strategy is outlined in Table 1 (please refer to Appendix 1 for details on the complete database-specific search strategies enacted in English, Spanish, and Portuguese). Following the database search, the reference lists of included studies were screened for other unidentified but potentially relevant studies.

**Table 1:** Simplified search strategy.

Domain	Transportation
MeSH	“Bicycling” [Mesh: focus] OR
Keywords	(Bike lane* OR bike way* OR bike path* OR bikeway* OR cicloruta* OR bicycl* OR cycling Bus rapid transit OR BRT OR Aerial lift* OR aerial tram* OR cable car* OR metrocable OR gondola lift* OR gondola car* OR cable propelled transit OR (Ciclovia*OR mass event* OR mega event* OR open street*).ti, ab.
	**Health**
MeSH	“Disease” [Mesh: focus] OR “Health” [Mesh: focus] OR “Urban Health” [Mesh: focus] OR “Public Health” [Mesh: focus] OR “Mortality” [Mesh: focus] OR “Wounds and Injuries” [Mesh: focus] OR
Keywords	(health OR disease* OR behavio* OR injur*OR fatal* OR mortalit*).ti, ab.
	**Study design**
MeSH	“Non-Randomized Controlled Trials as Topic” [Mesh: focus] OR “Follow-Up Studies” [Mesh: focus] OR “Controlled Before-After Studies” [Mesh: focus] OR
Keywords	(Systematic review* OR quasi-experiment* OR social experiment OR natural experiment* OR difference in difference* OR pre-post OR evaluation OR impact assessment* OR before and after OR Simulation OR systems model* OR agent-based model* OR multi-agent model* OR individual-based model* OR system dynamics).ti, ab.

ti = title; ab = abstract.

### 2.3 Study selection and inclusion criteria

The study selection process included three key steps. First, two reviewers (IS and LMTG) searched all relevant electronic databases in August and September 2017, imported search results into EndNote, removed all reference types other than journal articles and conference proceedings as well as papers in languages other than English, Spanish, or Portuguese. Second, all duplicate records were removed from EndNote using exact match for author and title. The remaining citations were then imported into Covidence (2017), a Cochrane review management platform. Additional duplicate citations identified within Covidence were also removed.

Second, the titles and abstracts of retrieved studies were screened by two pairs of independent reviewers within Covidence and assessed for inclusion in the review. Primary studies were selected for inclusion if they used empirical analyses or system-based simulation methods to evaluate the health impact of Open Streets programs, BRT, bicycle lanes, or aerial tram infrastructure. Health impact was defined broadly to include health outcomes or health-related behaviors such as bus, BRT and aerial tram use, bicycling, walking and more generic physical activity measures. Studies assessing mode share as an outcome were also included if they considered bus, BRT, bicycle and/or aerial tram use within their definition of mode share. We included these studies because these modes represent active forms of transportation and therefore qualify as health-related behaviors. Systematic reviews were not included, though they were used to help identify potentially relevant studies not identified directly through the search. While no limits were placed on participant demographics, where possible, the search was restricted to studies published in English, Spanish, and Portuguese. The Participants, Interventions, Comparators, Outcomes, and Study design (PICOS) approach was used to assess whether identified studies met the review's inclusion criteria (Table 2). Disagreements related to eligibility were resolved through discussions within reviewer pairs.

**Table 2:** PICOS criteria for study inclusion and exclusion.

INCLUDE	EXCLUDE
**Participants**	
Studies including participants of any age group	Animal studies
**Interventions**	
Studies evaluating the impact of new BRT, Open Streets programs, bicycle paths and/or aerial tram infrastructure on at least one health-related behavior and/or health outcome. Studies evaluating traffic-free bicycle infrastructure, such as multi-use trails, bridges and boardwalks that report on cycling outcomes separately. Multicomponent interventions, including those implemented across different sites, were included only if the impact of BRT, Open Streets programs, bicycle lanes and/or aerial trams was explicitly evaluated and reported on in at least one of these sites. Systems-based simulation studies exploring the impact of transportation mode choice (including bus, bicycle and/or aerial trams) on at least one health-related behavior and/or health outcome were included, even if no policy scenarios were simulated	Studies evaluating light rail transport systems, bicycle boxes, intersection crossings and roundabouts. System-based simulation studies exploring transportation choices that do not include bus, cycling or aerial trams, or do not consider health-related behavior or health outcomes. Simulation studies modelling route choice but not reporting on mode share including at least one of the aforementioned modes, or health-related behaviors or health outcomes.
**Comparators**	
Evaluations and system-based simulation studies comparing the impact of BRT, bicycle paths, Open Streets programs and aerial tram policies and/or transportation choices including at least one of these modes, on health-related outcomes.	
**Outcomes**	
Studies that report on at least one health-related behavior or health outcome, including injury, prevalence and counts of walking or cycling, time and distance walked or cycled (surrogates of physical activity energy expenditure (PAEE)), cycling speed (only if distance and/or time travelled are reported, thus enabling the assessment of PAEE). Studies reporting on mode share including bus, bicycle or aerial trams were also included.	Studies reporting on intermediary outcomes, such as air pollution, intentionality for behavior change or car crashes, without making a link to health-related behavior or health outcomes.
**Study design**	
Studies published in peer-reviewed journals or as peer-reviewed conference proceedings. Quasi-experimental studies and natural experiments with longitudinal designs. Systems-based simulation studies, such as agent-based models and system dynamics models	Studies that only collected follow-up data before the intervention was completed or that are cross-sectional in nature. Qualitative studies providing no quantitative assessment of policy effects and simulation studies that are not systems-based (e.g., health impact models). Commentaries and opinion pieces.

Third, full-text manuscripts of all potentially eligible studies were retrieved and screened by IS and LMTG. An interlibrary loan request was made for all manuscripts that could not directly be accessed. Studies that appeared to meet all the inclusion criteria of the review were discussed by the two reviewers and any disagreements were resolved by reflecting on the inclusion criteria and through consultation with the project working group. Systematic reviews and the reference lists of included studies were searched to identify any other potentially relevant papers.

### 2.4 Data extraction and analysis

Two data extraction tools (one for evaluations and another for system-based simulation studies) were developed to capture idiosyncratic aspects of study design and implementation characteristics of policy evaluations and simulation studies. Each tool was independently pilot tested on three studies and amended based on in-depth discussions with the project working group. The refined tool applied to evaluation studies extracted information on: author, year, geography, study type, aims or scope, sample size, participants, policy characteristics, length of follow-up, outcomes, the nature and significance of policy effects. The extraction tool applied to simulation-based studies sought to capture information relating to key aims or scope, software, and model specification – including, conceptual models, agent properties, actions and rules, temporal structure and characteristics of the model environment – modeled policy scenarios, outcomes, model parameters, calibration and validation processes, and key findings.

Five independent reviewers working in four pairs (FM & JDM, LMTG & MAM, IS & MAM and LMTG & IS) conducted an in-depth review of all included studies and performed extractions in accordance with the two extraction tools described above. The extractions were compared by each pair and discrepant information relating to any given study was discussed until all conflicts were resolved. Given the heterogeneity in study designs, exposures and outcomes reported by papers included in this review, a meta-analysis was not possible. Instead, a narrative synthesis of information extracted for each of the two extraction tool formats was conducted in accordance with Cochrane recommendations (Higgins et al., 2016).

### 2.5 Quality assessment

The quality of included studies was assessed using a modified version of the Newcastle-Ottawa Quality Assessment Scale for Cohort Studies (Wells et al., 2013). Information relating to intervention/policy characteristics, sampling strategy, representativeness of the target population, comparability of study controls and target populations, nature of outcome assessment, timing and adequacy of follow-up, and rates of attrition was collected from all evaluation studies. Given the absence of tools or guidance relating to the quality assessment of system-based simulation studies, we extracted information that we believed would provide a good indication of the quality of a given model's simulated output. This included information concerning sources used to inform model parameters, transparency concerning the equations and assumptions made, the presence of calibration, validation and sensitivity or uncertainty analyses.

## 3 Results

**Fig. 1:**
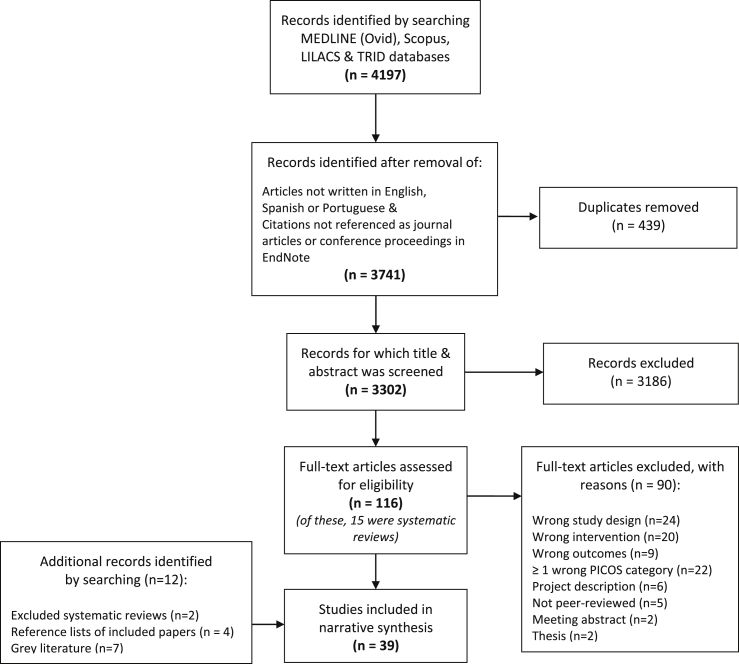
PRISMA flowchart showing process of study selection.

A total of 4197 records were identified by the search strategy implemented in each of the four databases; MEDLINE(Ovid), Scopus, LILACS and TRID (see Fig. 1). Records written in a language other than English, Spanish or Portuguese and those not referenced as journal articles or conference proceedings were removed, as were 439 duplicates. The remaining 3302 citations were screened for inclusion based on title and abstract. Most records (n = 3186) did not meet our inclusion criteria. We screened 116 full-text papers (including 15 systematic reviews) and excluded the majority of these (n = 90) for the reasons detailed in Fig. 1. The identified systematic reviews were all excluded after they were screened for potentially relevant primary studies. A total of 26 studies were identified using the employed search strategy. Thirteen additional studies were identified by: screening excluded systematic reviews (n = 2) and reference lists of included studies (n = 4), as well as by searching other sources (e.g., reviews and papers identified by the authorship team (n = 7)). Ultimately, a total of 39 studies were included in the systematic review, including 29 empirical studies and 10 simulation-oriented papers.

### 3.1 Empirical studies

Empirical studies included in the review were published between 2005 and 2017 (Table 3). These studies were based in: North America, including the United States (n = 11) (Boarnet et al., 2005; Evenson et al., 2005; Burbidge and Goulias, 2009; Parker et al., 2011; Chen et al., 2012; Parker et al., 2013; Dill et al., 2014; Brown et al., 2016; Cook et al., 2016; Ferenchak and Marshall, 2016; Brown et al., 2016b) and Canada (n = 1) (Bhatia et al., 2016); Europe, specifically, the United Kingdom (n = 9) (Goodman et al., 2013; Goodman et al. 2013b; Goodman et al. 2014; Heinen et al., 2015; Panter and Ogilvie, 2015; Heinen and Ogilvie, 2016; Panter et al., 2016; Panter and Ogilvie, 2017; Song et al., 2017) and Denmark (n = 1) (Jensen, 2008); and Australia (n = 4) (Greaves et al., 2015; Langdon, 2015; Rissel et al., 2015; Heesch et al., 2016). Only three studies were based in LMIC, specifically, Colombia, Mexico and Brazil (Cerdá et al., 2012; Pazin et al., 2016; Chang et al., 2017).

Of the 29 studies, 19 were single-component interventions focused on the implementation of bicycle lanes. The remaining 10 studies evaluated multicomponent interventions. Among these, one study evaluated newly built aerial tram infrastructure which was implemented in combination with other neighborhood improvements including, additional lighting in public spaces, pedestrian bridges and recreational centers (Cerdá et al., 2012). Five studies evaluated the health effects of bicycle lanes which were variously combined with a host of interventions such as light rail and pedestrian infrastructure improvements, the creation of parking facilities and additional lighting (Boarnet et al., 2005; Goodman et al., 2013; Brown et al., 2016; Pazin et al., 2016; Brown et al., 2016b). The remaining studies (n = 4) combined BRT and bicycle/pedestrian paths with park-and-ride sites (Heinen et al., 2015; Heinen and Ogilvie, 2016; Panter et al., 2016; Chang et al., 2017). Table 3 summarizes the characteristics of included studies.

**Table 3:** Characteristics of included evaluation studies.

ID	Author & year	Intervention location & period	Intervention type	Intervention description/definition of population and groups	Population/groups (n)	Baseline age (years)	Female (%)	Outcome Type(s) analyzed	Outcomes stratified; groups
1	Bhatia et al. (2016)	Toronto, CAN 1993–2008	SC: cycling infrastructure; bike lanes (unprotected)	Seven lane segments in which a bicycle lane was painted between 1991 and 2010. The lane segments also had a higher collision cycle lane (i.e., Minimum of 100 cycle-motor vehicle collisions between 1991 and 2010). **Population:** Cycle-motor vehicle collisions reported on the 7 lane segments with newly marked bike lanes	Mainly Adults Collisions (329)	Unclear	Unclear	Injury (n = 3)	No
2	Boarnet et al. (2005)	Murrieta, USA unclear	MC: cycling infrastructure; bike lanes (unprotected)	Bicycle facilities including on-street bike lanes were installed at one site i.e., at Murrieta Elementary. “New sidewalks and sidewalk gap closures” were also constructed around the school. [p.308] **Population:** Adult and child cyclists using the new bike lanes	Combined adults & children Cyclists/Pre (4) Post (14)	Unclear	Unclear	Active transit trips (n = 1)	No
3	Brown et al. (2016)	Salt Lake City, USA 2013	MC: cycling infrastructure; bike lanes (unclear)	The complete streets intervention included the completion of a sporadic bike lane and involved widening sections to make it a designated “high comfort” bike lane (with speed limit reduced to 50 km/h) on the city bike map. The bike lanes were painted on both sides of the street and the sidewalks were improved and widened to create a shared bike and pedestrian path. The intervention also included a light rail line extension, narrowing of automotive lanes and the creation of wider and better lit sidewalks. **Intervention (near) group:** Adults living (≤800 m) from the complete street renovation **Control (far) group:** Adults living 801–2000m from complete street renovation	Adults (536)	NR	51	Active transit mode share (n = 3)	No
4	Brown et al. (2016b)	Salt Lake City, USA 2013	MC: cycling infrastructure; bike lanes (unclear)	The complete streets intervention included the completion of a sporadic bike lane and involved widening sections to make it a designated “high comfort” bike lane (with speed limit reduced to 50 km/h) on the city bike map. The bike lanes were painted on both sides of the street and the sidewalks were improved and widened to create a shared bike and pedestrian path. The intervention also included a light rail line extension, narrowing of automotive lanes and the creation of wider and better lit sidewalks. **Population:** Adults living within 2 km of the complete streets intervention. Participants were divided into four cycling groups based on their cycling patterns pre and post intervention: *never cyclists* (not cycled pre or post) which serve as the reference group; *continuing cyclists* (cycled pre and post); *former cyclists* (cycled pre but not post); *new cyclists* (not cycled pre but cycled post).	Adults			Physiological (n = 2) Anthropometric (n = 1) Active transit duration (n = 1)	No
					**Intervention**				
					Cyclist group:				
					Never (434)	43	55		
					Continuing (29)	40	17		
					Former (33)	38	30		
					New (40)	37	43		
5	Burbidge and Goulias (2009)	Salt Lake City, USA 2007	SC: cycling infrastructure; multi-use trail (separated)	“A Class 1 trail (two-way multi-use trail separated from existing roads and sidewalks) [was constructed] on the existing canal right-of-way.” “This trail creates a 4.025 km loop connecting two existing sidewalks.” [p.79] **Population:** Adult residents of West Valley City, a suburb of Salt Lake City, who live within 1.6 km of the trail	Adults (98)	48	55	Active transit trips (n = 3) Active transit duration (n = 1)	Yes; age
6	Cerdá et al. (2012)	Medellin, COL 2004	MC; aerial trams	A cable-propelled transit system (gondola) known as Metrocable was built using funding from the municipal government of Medellin as part of “a territorial plan to promote urban and rural development”. It connects “an elevated train system in the city centre to the impoverished Santo Domingo neighborhood in the mountainous periphery, with 4 stops covering a distance of 2072 m and reaching an elevation of 399 m” [p.1046] “The municipal government made other improvements to neighborhoods serviced by the gondola, including additional lighting for public spaces; new pedestrian bridges and street paths; ‘‘library parks’’; buildings for schools, recreational centers, and centers to promote microenterprises; more police patrols; and a family police station next to a gondola station.” [p.1046] **Intervention group:** Residents (12–16 years) of one of 25 neighborhoods (city districts 1 and 2) where the gondola system was installed. **Control group:** Residents of one of 23 neighborhoods located in comparable city districts (4 and 8) that were not serviced by the gondola system.	Adolescents & Adults	36–61 yrs:		Homicide (n = 1)	No
					**Intervention** (225)	26%	67		
					**Control** (241)	27%	67		
7	Chang et al. (2017)	Mexico City, MEX 2013	MC: BRT & cycling infrastructure; bike lanes (separated)	The Metrobus Line 5 corridor was added to an existing BRT network with four existing lines in Mexico City. The Line 5 corridor is 10 km long with 18 stations, with an average of 625 m between stations. Its service features include: “(i) articulated and bi-articulated high floor buses, (ii). Exclusive bus lanes, and (iii) off-board fare collection” [p.339]. The BRT intervention was combined with a Complete Street intervention which included a host of streetscape interventions including protected bike lanes and parking, widened sidewalks, redesigned junctions, and the recovery of public and green space throughout the corridor. **Population**: Adults residing 500 m either side of the Line 5 corridor.	Adults	Mean age:		Active transit frequency (n = 3)	Yes; gender, education, employment type
					**Intervention** (1420)	47	52		
					**Control** (1067)				
8	Chen et al. (2012)	New York City, USA 1996–2006	SC: cycling infrastructure; bike lanes (unprotected)	Intervention included the creation of 69 km of bicycle lanes on 61 streets not protected by a parking lane, in the 5 boroughs of New York City from 1996 through 2006. **Intervention group:** Road users in New York City travelling on roadways where on-street bicycle lanes (not protected by a parking lane) had been installed from 1996 through 2006 (a total length of about 69.2 km on 61 streets). **Control group:** Road users travelling on roadways without bicycle lanes but with segment- or intersection-level characteristics comparable to those of the treatment group.	Unclear Cyclists **Intervention** Pre (4360) Post (1349) **Control** Pre (12,365) Post (3578)	Unclear	Unclear	Injury (n = 2)	No
9	Cook et al. (2016)	Durham, USA 2014	SC: cycling infrastructure; multi-use trail (separated)	A 3.2 km long bicycle and pedestrian bridge-link was created to connect the northern segment (11.3 km long) of the trail to the southern trail segment (21.7 km long) to form a continuous 35 km shared use, separated path. **Population**: Users of the trail segments both to the north and south of the bridge linkage	Unclear Trail users Pre (1301 survey; 9266 counts) Post (2245 survey; 21,365 counts)	26–54 yrs	45	Active transit mode share (n = 4) Active transit duration (n = 4)	Yes; household income
10	Dill et al. (2014)	Portland, USA unclear	SC: cycling infrastructure; bike lanes (unprotected)	A new bicycle boulevard was installed on 8 street segments (1.45 km–6.76 km long) in Portland, Oregon. Eleven control street segments (1.6–9.2 km long) were also monitored as part of the evaluation. **Intervention group:** Adults residing within 300 m the 8 streets selected for bicycle boulevard installation. **Control group:** Adults residing within 300 m of the 11 control street segments.	Adults			Active transit trips (n = 1) Active transit mode share (n = 3) Active transit duration (n = 2) Physical activity (n = 1)	No
					**Intervention** (182)	43	63		
					**Control** (168)	41	67		
11	Evenson et al. (2005)	Durham, USA 2002	SC: cycling infrastructure; multi-use trail (separated)	This study evaluated a railway track segment that was converted to a paved, 3-m-wide multi-use trail, which extended an existing 5.1 km trail segment by another 4.5 km, along with a 3.2 km spur. **Population:** Adults living within 3.2 km of the new trail segment.	Adults (366)	43% ≥50 yrs	65	Physical activity (n = 5) Active transit duration (n = 3)	No
12	Ferenchak and Marshall (2016)	Chicago, USA 2008-10	SC: cycling infrastructure; bike lanes & sharrows (both unprotected)	259 block groups that had only sharrows (shared lane markings) installed (overall 54 km of sharrows), and 292 block groups that overall had 168 km of bike lanes installed. **Intervention group:** *Bike lane*; census block groups that had bike lanes or trails installed between 2000 and 2010. *Sharrow group;* census block groups that had only sharrows installed between 2000 and 2010. **Control group:** Block groups that had no bike infrastructure installed between 2000 and 2010.	Unclear Cyclists **Intervention***Bike lane* (1621 ridership; 2046 safety outcome) *Sharrow* (259 ridership; 89 safety outcome) **Control** (292 ridership; 39 safety outcome)	Unclear	Unclear	Active transit trips (n = 2) Active transit mode share (n = 2) Injury (n = 2)	No
13	Goodman et al. (2013)	18 towns^a^, GBR 2005-11	MC: cycling infrastructure; bike lanes (unprotected) & bike tracks (protected)	The town-level initiatives involved mixtures of capital investment (e.g. cycle lanes) and revenue investment (e.g. cycle training), tailored to each town. “Each town implemented a different mixture of infrastructure, tailored to its specific context. In total, 98 km of on-road lanes and 264 km of off-road paths were created between 2008 and 2011. This represented a 28% increase in the length of such routes previously available (based on 16 of 18 towns reporting sufficient data on pre-intervention facilities)” [p.230]. The capital investment component involved an increase in cycle lanes and paths ranging from as low as 9% (Stoke-on-Trent) to as high as 105% in Brighton & Hove. **Intervention group:** 17 urban towns and one city outside of London selected to be part of initiative. **Comparison groups:***Matched comparison*; “largest urban regions within the English local authority ‘most similar’ similar’ to each intervention local authority.” Similarity was based on demographic, socioeconomic, employment and industry characteristics. *Unfunded comparison*; “largest urban region within the 67 local authorities which applied unsuccessfully” for the initiative. *National comparison*; “all non-intervention, urban areas outside London with a population of >30,000 (close to the size of the smallest intervention town)” [p.231].	Adults **Intervention group** (1,266,337) **Comparison group** *Matched comparison* (969,605) *Unfunded comparison* (4,195,540) *National comparison* (10,356,452)	Unclear	Unclear	Active transit mode share (n = 3)	Yes; area-level deprivation
14	Goodman et al. (2013b)	Cardiff, Kenilworth & Southampton, GBR 2010-11	SC: cycling infrastructure; bike lanes (separated)	Three Connect2 projects were evaluated. These were based in Cardiff, where a new 140 m long, 4 m wide traffic-free bridge with integral lighting was built over Cardiff Bay; Kenilworth, where a traffic-free bridge was built over a busy trunk road to link the town to a rural greenway; and Southampton, where an informal riverside footpath (impassable at high tide) was turned into a new 400 m boardwalk. **Population:** Adults residing within 5 km by road network from the core Connect2 projects in each of the three towns	Adults	≥50 yrs		Active transit mode share (n = 4)	Yes; education, income
					**Intervention**				
					1-year follow-up sample (1849)	66%	54		
					2-year follow-up sample (1510)	70%	57		
15	Goodman et al. (2014)	Cardiff, Kenilworth & Southampton, GBR 2010-11	SC: cycling infrastructure; bike lanes (separated)	Three Connect2 projects were evaluated. These were based in Cardiff, where a new 140 m long, 4 m wide traffic-free bridge with integral lighting was built over Cardiff Bay; Kenilworth, where a traffic-free bridge was built over a busy trunk road to link the town to a rural greenway; and Southampton, where an informal riverside footpath (impassable at high tide) was turned into a new 400 m boardwalk. **Population:** Adults residing within 5 km by road network from the core Connect2 projects in each of the three towns	Adults	≥50 yrs		Active transit duration (n = 6) Physical activity (n = 1)	No
					**Intervention**				
					1-year follow-up sample (1796)	66%	56		
					2-year follow-up sample (1465)	70%	57		
16	Greaves et al. (2015)	Sydney, AUS 2014	SC: cycling infrastructure; bike tracks (separated)	“The intervention comprised a 2.4 km length of separated bi-directional cycleway linking the inner-city suburbs of Green Square in the south with the Central Business District (CBD) through Redfern and Waterloo”. “The George Street cycleway adds to several pre-existing bi-directional cycleways within the City of Sydney Local Government Area (LGA) totaling a distance of 11 km (as of October 2014)” [p.4]. **Intervention group**: Geographic area encompassing new cycleway in inner Sydney **Control group:** Neighboring area of similar demographics with no new planned bicycle infrastructure	Adults	45–55 yrs		Active transit trips (n = 5) Active transit duration (n = 1) Active transit time share (n = 1)	No
					**Intervention** (184)	46%	61		
					**Control** (251)	unclear	unclear		
17	Heesch et al. (2016)	Brisbane, AUS 2013	SC: cycling infrastructure; bike tracks (separated)	“The V1 is a dedicated 17-km long, 3-m wide exclusive off-road bikeway.” “The V1 has been delivered in stages with Stage A (about 1.4 km) completed in July 2010, and Stage B (about 900 m) completed in May 2011. Completion of these stages extended existing V1 bikeway infrastructure farther south. Stage C (about 2.3 km) is 7 km north of these earlier improvements via the existing V1 infrastructure. It opened June 25, 2013 to extend the V1 farther north towards the city centre” [p.368]. **Intervention group:** Cyclists on the newly created bikeway i.e., the Veloway 1 (or V1). **Reference group:** Cyclists on the South East Freeway Bikeway (or SEFB).	Unclear			Active transit trips (n = 1)	No
					Cyclists	Unclear	15		
					**Intervention** (169)				
					**Reference**				
					Pre (132)	Unclear	14		
					Post (99)	Unclear	20		
18	Heinen and Ogilvie (2016)	Cambridge, GBR 2011	MC: cycling infrastructure; multi-use trail (separated) & BRT	“The busway comprises a 25 km off-road guideway for buses, with a parallel path that can be used for walking and cycling, in two sections: one between the market town of St Ives and the northern edge of Cambridge, and the other between Cambridge railway station and the southern fringe at Trumpington” [p.2]. It also includes three park-and-ride sites. **Intervention group:** Adults aged 16 or over, working in areas of Cambridge to be served by the busway and living within approximately 30 km of the city centre.	Adults (470)	≥51 yrs 34%	67	Active transit mode share (n = 10)	No
19	Heinen et al. (2015)	Cambridge, GBR 2011	MC: cycling infrastructure; multi-use trail (separated) & BRT	“The busway comprises a 25 km off-road guideway for buses, with a parallel path that can be used for walking and cycling, in two sections: one between the market town of St Ives and the northern edge of Cambridge, and the other between Cambridge railway station and the southern fringe at Trumpington. It also includes three park-and-ride sites” [18, p.2]. **Intervention group:** Adults aged 16 or over, working in areas of Cambridge to be served by the busway and living within approximately 30 km of the city centre.	Adults (466)	≥51 yrs 34%	67	Active transit mode share (n = 6)	No
20	Jensen (2008)	Copenhagen, DNK 1978–2003	SC: cycling infrastructure; bike lanes (unprotected) & bike tracks (separated)	Construction of one-way bicycle track (2–2.5 m wide) on both sides of a 20.6 km road and marking of one -way bicycle lanes (1.5–2 m wide) on both sides of a 5.6 km road in Copenhagen, Denmark. **Intervention group:** Collisions on roads with newly constructed bike lanes and tracks. **General comparison group:** Collisions on unchanged roads with known developments in traffic volume. This group consists of 110 km of roads with 170 locations, where motor vehicle and bicycle/moped traffic is counted yearly or every forth to sixth year.	Unclear Collisions **Intervention** *On Bike lanes:* Crashes (700); Injuries (219). *On Bike tracks:* Crashes (5,898); Injuries (2,413). **Comparison** Crashes (24,369) Injuries (8,648)	Unclear	Unclear	Injury (n = 30)	No
21	Langdon (2015)	Brisbane, AUS 2001-10	SC: cycling infrastructure; bike lanes (unclear)	The intervention included the installation of “bridges, missing links and end of trip facilities for cyclists” [p.1]. The bridges evaluated include the: Goodwill Bridge 2001; Go-between Bridge & Kurilpa Bridge; Eleanor Schonell Bridge; Sir Leo Hielsher (Gateway) Bridge; and the Ted Smout Memorial Bridge. The missing links included the: Normanby Pedestrian Cycle Link; Western Freeway Bikeway & Toowong Overpass; and the Veloway 1 (V1) Stage C. **Population:** Bicycle commuters on the bridges and along missing links installed.	Unclear Cyclists (8,600)	Unclear	Unclear	Active transit mode share (n = 1)^b^ Active transit trips (n = 1)^b^	No
22	Panter and Ogilvie (2015)	Southampton, Cardiff & Kenilworth, GBR 2010-11	SC: cycling infrastructure; bike lanes (separated)	Three Connect2 projects were evaluated. These were based in Cardiff, where “a new 140 m long, 4 m wide traffic-free bridge with integral lighting” was built over Cardiff Bay; Kenilworth, where “a traffic-free bridge was built over a busy trunk road to link the town to a rural greenway”; and Southampton, where an informal riverside footpath (impassable at high tide) was turned into “a new 400 m boardwalk” [p.2]. **Population:** Adults residing within 5 km by road network from the core Connect2 projects in each of the three towns	Adults (967)	≥50 yrs 65%	52	Active transit duration (n = 1)	No
23	Panter et al. (2016)	Cambridge, GBR 2011	MC: cycling infrastructure; multi-use trail (separated) & BRT	“The busway comprises a 25 km off-road guideway for buses, with a parallel path that can be used for walking and cycling, in two sections: one between the market town of St Ives and the northern edge of Cambridge, and the other between Cambridge railway station and the southern fringe at Trumpington. It also includes three park-and-ride sites” [18, p.2]. **Intervention group:** Adults aged 16 or over, working in areas of Cambridge to be served by the busway and living within approximately 30 km of the city centre.	Adults (469)	Mean age: 44	67	Physical activity (n = 2) Active transit trips (n = 6)	No
24	Panter and Ogilvie (2017)	Southampton, Cardiff & Kenilworth, GBR 2010-11	SC: cycling infrastructure; bike lanes (separated)	Three Connect2 projects were evaluated. These were based in Cardiff, where “a new 140 m long, 4 m wide traffic-free bridge with integral lighting” was built over Cardiff Bay; Kenilworth, where “a traffic-free bridge was built over a busy trunk road to link the town to a rural greenway”; and Southampton, where an informal riverside footpath (impassable at high tide) was turned into “a new 400 m boardwalk” [22, p.2]. **Population:** Adults residing within 5 km by road network from the core Connect2 projects in each of the three towns	Adults (1258)	≥50 yrs 72%	55	Active transit trips (n = 6)	No
25	Parker et al. (2011)	New Orleans, USA 2008	SC: cycling infrastructure; bike lanes (unprotected)	“The 5.0 km dedicated bike lane on St. Claude Avenue, also known as Louisiana Highway 46”, was completed in the spring of 2008. “Bike lanes were striped on both sides of the road and are 1.5 m wide” [p.S99]. **Population:** Adults and children cycling along St. Claude Avenue.	Combined adults & children	NR	Mean number	Active transit trips (n = 1)	Yes; gender
					Cycling trips				
					**Intervention**				
					Pre (mean: 121)		(13)		
					Post (mean: 188)		(29)		
26	Parker et al. (2013)	New Orleans, USA 2010	SC: cycling infrastructure; bike lanes (unprotected)	“The 1.6 km dedicated bike lane on S. Carrollton Avenue”, New Orleans, completed in June 2010. “Bike lanes were striped on both sides of the road and are 1.5 m wide. There is one 3.4 m wide travel lane on either side of the road, separated by a 18 m wide median” [p.S102]. **Intervention group:** Adults and children cycling along S. Carrollton Avenue **Comparison group:** Adults and children cycling on two adjacent side streets; Short and Dublin St.	Combined adults & children	NR	Mean number	Active transit trips (n = 1)	Yes; race, gender
					Cycling trips				
					**Intervention**				
					(mean: 257)		(15)		
					**Comparison**				
					(mean: 37)		(33)		
27	Pazin et al. (2016)	Florianópolis, BRA 2010	MC: cycling infrastructure; bike lanes (unprotected)	A new walking and cycling route (2.3 km long) was inaugurated in [an area known as Beira-Mara Continental,] in the continental coast of Florianópolis, SC, Brazil.” “The project included a new avenue, parking lots, and an on-road walking and cycling route, all along the seashore” [p.19]. **Population:** Adult residing within 0–500 m, 501–1000 m and 1001–1500 m of the new walking and cycling route.	Adults (519)	55–85 yrs:		Physical activity (n = 4) Active transit mode share (n = 1) Active transit frequency (n = 1)	No
					**Intervention**	41%	58		
					*0-500m* (192)	43%	55		
					*501-1000m* (137)	46%	54		
					*1001-1500m* (190)	37%	65		
28	Rissel et al. (2015)	Sydney, AUS 2014	SC: cycling infrastructure; bike track (separated)	“New 2.4 km bi-directional separated bicycle path built” as part of its expanding bicycle network in Sydney [p.1]. **Intervention group:** Adults living no more than 2.5 km from the new bicycle path. **Comparison group:** Adults living in “neighborhoods a similar distance from the central business district and with a similar demographic profile, and where local council had no plans to modify infrastructure during the study period” [p.2].	Adults (512) **Intervention** (240) **Comparison** (272)	≥45 yrs: 37%	63	Active transit trips (n = 2) Active transit frequency (n = 1)	No
29	Song et al. (2017)	Cardiff, Kenilworth & Southampton, GBR 2010-11	SC: cycling infrastructure; bike lanes (separated)	Three Connect2 projects were evaluated. These were based in Cardiff, where “a new 140 m long, 4 m wide traffic-free bridge with integral lighting” was built over Cardiff Bay; Kenilworth, where “a traffic-free bridge was built over a busy trunk road to link the town to a rural greenway”; and Southampton, where an informal riverside footpath (impassable at high tide) was turned into “a new 400 m boardwalk” [22, p.2]. **Population:** Adults residing within 5 km by road network from the core Connect2 projects in each of the three towns	Adults **Intervention** (1489)	57	56	Active transit distance (n = 6) Active transit distance share (n = 2) Active transit duration (n = 6) Active transit time share (n = 2) Non-active transit distance (n = 2) Non-active transit duration (n = 2)	No

SC: Single component intervention; MC: Multicomponent intervention; NR: not reported; CAN: Canada; USA: United States of America; DNK: Denmark; GBR: United Kingdom; AUS: Australia; BRA: Brazil; COL: Colombia; NOTE: For multicomponent interventions, this table reports information relating to the interventions relevant to our review, and the population characteristics and outcomes assessed only for relevant interventions.

a 18 towns: Darlington, Derby, Brighton & Hove, Aylesbury, Exeter, Lancaser with Morecambe, York, Cambridge, Colchester, Southend-on-Sea, Leighton Buzzard, Woking, Bristol, Shrewsbury, Stoke-on-Trent, Chester, Southport & Ainsdale, Blackpool; *CAN* Canada, *USA* United States of America, *DNK* Denmark, *GBR* United Kingdom, *AUS* Australia, *BRA* Brazil, *COL* Colombia *NOTE: For multicomponent interventions, this table reports information relating to the interventions relevant to our review, and the population characteristics and outcomes assessed only for relevant interventions*.

b Outcomes not tested for statistical significance.

All 29 empirical evaluation studies employed a longitudinal study design and included pre- and post-intervention observations. Of these 29 studies, 15 included a control or reference group (Jensen, 2008; Cerdá et al., 2012; Chen et al., 2012; Goodman et al., 2013; Parker et al., 2013; Dill et al., 2014; Greaves et al., 2015; Panter and Ogilvie, 2015; Rissel et al., 2015; Brown et al., 2016; Heesch et al., 2016; Pazin et al., 2016; Brown et al., 2016b; Panter and Ogilvie, 2017; Song et al., 2017). The proportion of studies with a control or reference group did not differ by intervention type; around half of all single component (10 of 19 studies) and multi-component intervention (5 of 10 studies) reported either a control or reference group. Four studies employed a random sampling approach to recruit participants, including three multi-component (Cerdá et al., 2012; Pazin et al., 2016; Chang et al., 2017) and one single component intervention study (Evenson et al., 2005). A further eleven studies attempted to sample the entire study area, including nine single component (Chen et al., 2012; Goodman et al. 2013b, 2014; Dill et al., 2014; Panter and Ogilvie 2015, 2017; Bhatia et al., 2016; Ferenchak and Marshall, 2016; Song et al., 2017) and two multi-component intervention studies (Goodman et al., 2013; Brown et al., 2016).

Exposure to the transportation intervention was operationalized in several different ways among the 29 empirical studies. Some studies compared geographic locations, specifically, street segments, before and after an intervention (Boarnet et al., 2005; Jensen, 2008; Parker et al. 2011, 2013; Chen et al., 2012; Bhatia et al., 2016; Cook et al., 2016; Heesch et al., 2016). Most studies however, considered intervention impacts among people living in a fixed area of influence. These areas of influence were most commonly defined as buffers centred on the focal point of an intervention (Evenson et al., 2005; Burbidge and Goulias, 2009; Dill et al., 2014; Panter and Ogilvie, 2015; Rissel et al., 2015; Brown et al., 2016b; Chang et al., 2017), although some studies used predefined geographies such as towns (Goodman et al., 2013), block groups (Langdon, 2015; Ferenchak and Marshall, 2016) and neighborhoods (Cerdá et al., 2012) in and/or surrounding intervention sites. Only 9 studies used a distance-based metric to assess health impacts among people living different distances from an intervention (Goodman et al. 2013b, 2014; Heinen et al., 2015; Brown et al., 2016; Heinen and Ogilvie, 2016; Panter et al., 2016; Pazin et al., 2016; Panter and Ogilvie, 2017; Song et al., 2017). We were unable to determine how exposure to the intervention was defined in one study (Greaves et al., 2015).

**Fig. 2:**
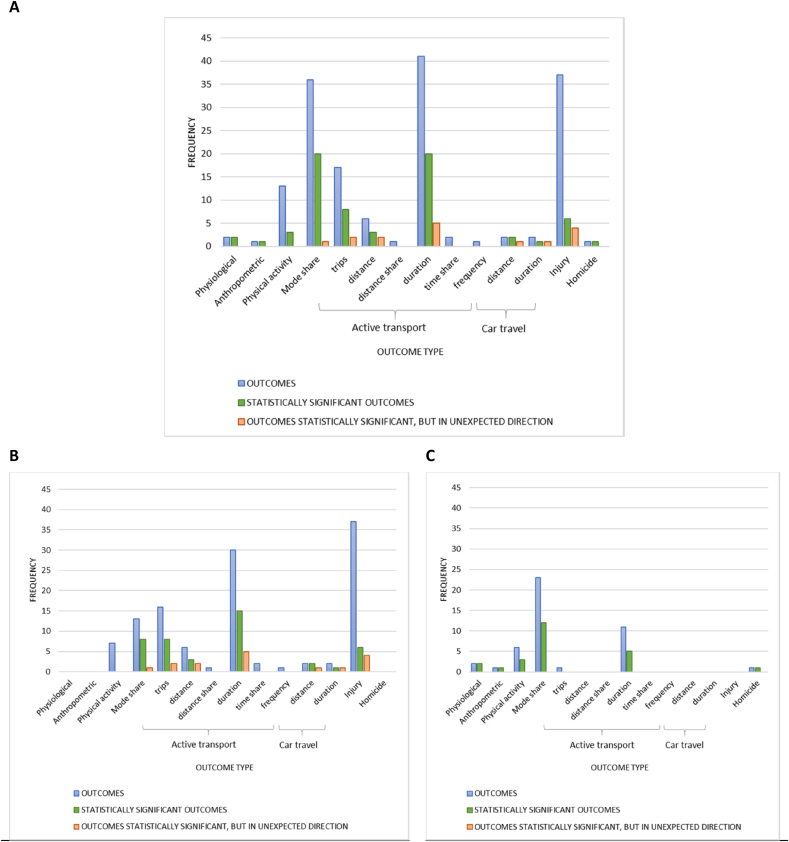
Frequency of outcomes, by type, for **A** all included empirical studies (29 studies, 162 distinct outcomes), **B** single component interventions (19 studies, 117 outcomes) focusing on the creation of bicycle lanes, and **C** multicomponent interventions (10 studies, 45 outcomes). The bars in blue represent the total number of outcomes, by type, while the green bars represent the total number of statistically significant outcomes. Of the statistically signficant outcomes, the bar in orange represents the number of outcomes that were in the unexpected direction for each outcome type. (For interpretation of the references to colour in this figure legend, the reader is referred to the Web version of this article.) NB: Figures do not include 21 outcomes reported by 5 studies because these outcomes were not assessed using statistical models.

A total of 183 outcomes relating to 19 unique interventions were assessed by the 29 empirical studies included in our review. Of these, 21 outcomes were described but statistical tests were not conducted (see Appendix 2) (Burbidge and Goulias, 2009; Dill et al., 2014; Greaves et al., 2015; Langdon, 2015; Song et al., 2017). The 162 outcomes tested for statistical significance varied both with respect to outcome type and the frequency with which they were evaluated (Fig. 2A; Appendix 3, Table 1). The four most frequently assessed outcome types included, active travel duration (25%), injury (23%), mode share (i.e., the share of walking, cycling, public transport and or car trips) (22%), and number of active trips (including walking or cycling trips) (11%). Few studies assessed physiological (Brown et al., 2016b), anthropometric (Brown et al., 2016b), and car travel-related outcomes (Greaves et al., 2015; Song et al., 2017). The three studies that evaluated the health effects of interventions in LMIC focused on homicide in Colombia (n = 1) (Cerdá et al., 2012), active transit frequency in Mexico and Brazil (n = 3 and n = 1, respectively) (Pazin et al., 2016; Chang et al., 2017), as well as physical activity (n = 4) and active mode share (n = 1) in Brazil (Pazin et al., 2016). Appendix 2 provides further information on assessed outcomes and the nature of the associations reported by included studies.

The 19 studies reporting on single-component interventions (all focusing on bicycle lanes; Fig. 2B) predominantly reported on injury outcomes (n = 37) (Jensen, 2008; Chen et al., 2012; Bhatia et al., 2016; Ferenchak and Marshall, 2016), active travel duration outcomes (n = 30) (Evenson et al., 2005; Burbidge and Goulias, 2009; Dill et al., 2014; Goodman et al., 2014; Greaves et al., 2015; Panter and Ogilvie 2015, 2017; Cook et al., 2016; Song et al., 2017) and active trip outcomes (n = 16) (Burbidge and Goulias, 2009; Parker et al. 2011, 2013; Dill et al., 2014; Greaves et al., 2015; Rissel et al., 2015; Ferenchak and Marshall, 2016; Heesch et al., 2016). Only 6 out of 37 injury outcomes showed statistically significant associations with new bicycle lanes (Jensen, 2008; Bhatia et al., 2016; Ferenchak and Marshall, 2016), and of these, the majority (n = 4, all from the same study) were in the unexpected direction (i.e., the installation of bicycle lanes was positively associated with injuries) (Jensen, 2008). The highest number of statistically significant outcomes were observed for active travel duration (i.e., 15 of 30 outcomes) (Evenson et al., 2005; Dill et al., 2014; Goodman et al., 2014; Cook et al., 2016; Panter and Ogilvie, 2017; Song et al., 2017). Of these 15 outcomes, 10 showed statistically significant associations in the expected direction, that is, active travel duration increased following the bicycle lane intervention, whereas the inverse was observed in five outcomes. The second highest number of statistically significant associations were observed for active trips (i.e., 8 of 16) (Burbidge and Goulias, 2009; Parker et al. 2011, 2013; Greaves et al., 2015; Rissel et al., 2015; Ferenchak and Marshall, 2016; Heesch et al., 2016) and active mode share (i.e., 8 of 14) (Goodman et al., 2013b; Cook et al., 2016; Ferenchak and Marshall, 2016). Of these, seven active mode share and six active trip outcomes were in the expected direction (i.e., the share of travel made using active modes and the number of active trips increased following the creation of new bicycle lanes).

Four single component intervention studies assessed the health impacts of living different distances from the site of the intervention. Two of these studies, found positive associations between proximity to cycling infrastructure and the duration of active travel (for 7 of 12 outcomes) (Goodman et al., 2014; Panter and Ogilvie, 2017) as well as the share of trips made using active modes (n = 4 of 4 outcomes) (Goodman et al., 2013b).

The ten studies reporting on multicomponent interventions (Fig. 2C) included a study evaluating the impact of aerial tram infrastructure on homicide (Cerdá et al., 2012), five studies evaluating bicycle lanes, and four studies focused on combined BRT and bicycle lane interventions (Heinen et al., 2015; Heinen and Ogilvie, 2016; Panter et al., 2016; Chang et al., 2017). Among the multicomponent interventions, one study (Brown et al., 2016b) uniquely reported on physiological and anthropometric outcomes such kilocalorie expenditure and body mass index. The most frequently reported outcome among multicomponent interventions was active travel mode share with just over half of these outcomes (i.e., 12 of 23) demonstrating a statistically significant and positive association with the installation of bicycle lanes and/or BRT infrastructure (Heinen et al., 2015; Brown et al., 2016; Heinen and Ogilvie, 2016; Pazin et al., 2016).

Of the 10 multicomponent interventions, five investigated whether living different distances from the intervention differentially impacted health outcomes. These five studies found that people living closer to bicycle lanes, with or without an adjoining BRT line, had higher levels of physical activity (for 3 of 6 outcomes) (Pazin et al., 2016), engaged in longer periods of active travel (for 3 of 7 duration outcomes) (Panter et al., 2016; Pazin et al., 2016), and used a higher share of active modes (for 9 of 20 outcomes) (Heinen et al., 2015; Brown et al., 2016; Heinen and Ogilvie, 2016; Pazin et al., 2016).

### 3.2 Simulation studies

The 10 system-based simulation studies (Table 4) included in this review (McDonnell and Zellner, 2011; Okushima and Akiyama, 2011; Yang and Diez-Roux, 2013; Macmillan et al., 2014; Okushima 2015, 2016; Yang et al., 2015; Lemoine et al., 2016a; Zellner et al., 2016; Zou et al., 2016) were published between 2011 and 2016.

**Table 4:** Characteristics of system-based simulation studies.

ID	Author & year	Modelling method	Intervention type	Setting modeled	Model description	Scenarios modeled	Relevant outcomes	Key findings
30	Lemoine et al. (2016a)	ABM	BRT	Stylized Bogota, Colombia	Agents: Adults Interactions: Agent-environment only Main processes: Agents choose the mode of transportation based on total trajectory costs and time needed to reach their workplace; the resource cost of each trip is deducted from a daily resource budget Time step: 1 day Period modeled: 40 days (only last 30 used for analysis)	Type 1 (6 scenarios): Increasing number of BRT lanes by substituting existing bus lanes; Type 2 (7 scenarios): Changing BRT and bus density	Walking for transportation	Increasing the number of BRT lanes increases minutes walked for transportation, observable also among nonusers of the BRT; walking time saturates as BRT lanes are added to the system
31	Macmillan et al. (2014)	System dynamics	Bikeways	Auckland, New Zealand	Main variables: Cyclists, normality of cycling, cyclist injuries, safety in numbers, real and perceived risk of injury, investment in cycle-friendly infrastructure, car commuters, average car speed, vehicles on the road at commute time Interactions: Person-person and person-environment Main processes: Balancing Causal Loop 1: Very little change in bicycling infrastructure combined with more people bicycling to work results in more bicycle crashes, influencing perceived risk of injury and reducing bicycling. Balancing Causal Loop 2: Significant shift from cars to bicycles at peak times results in faster vehicles and increased real and perceived risk of bicycling injury, deterring cyclists. Reinforcing Causal Loop 1: As the number of cyclists increases, their political influence rises, thereby increasing investment in safer bicycling facilities and encouraging bicycling. Reinforcing Causal Loop 2: More cycling to work can influence the behavior of other road users toward cycling. Reinforcing Causal Loop 3: Significant mode shift from cars to bicycle commutes results in reduced vehicle numbers and, therefore, lower likelihood of collision and higher perceptions of bicycle safety Time step: 1 year Period modeled: 1991 to 2051 (historical simulation from 1991 to 2012)	Type1 (1 scenario): Business as usual; Type 2 (6 scenarios): Regional Cycle Network (development of a partial network of mixed cycling infrastructure); Type 3 (6 scenario): Arterial segregated bicycle lanes (ASBL - gradual implementation of a one-way physically segregated lane on each side of every arterial road); Type 4 (6 scenarios): Self-explaining roads (SER - gradual transformation of all local roads into low-speed streets using nontraditional, endemic road features, such as street narrowing, trees, and art; Type 5 (12 scenarios): Combination of ASBL and SER interventions	Injury, physical activity, all-cause mortality	Benefits of all the intervention policies outweighed harms between 6 and 24 times; most effective approach would involve physical segregation on arterial roads and low speed, bicycle-friendly local streets; greatest benefits accrued from reduced all-cause mortality due to physical activity
32	McDonnell and Zellner (2011)	ABM	BRT	Stylized setting (generic single-direction 5-km road running to a destination representing a central business district)	Agents: Households (car-using household or bus-using household), bus riders, cars, buses, car entrances, bus stops Interactions: Agent-agent and agent-environment Main processes: Each household produces either bus riders or car users according to their modal choice; mode choice is represented as a decision to switch from bus to car or vice-versa when the travel time exceeds their tolerance for lengthening commutes; journey time of each agent depends on the presence of other agents on the road; car users on the road check the road patch directly in front of them and wait until a space becomes free; bus riders need to wait for the bus; buses start departing as per the frequency parameter; buses check for the presence of bus riders and their capacity; if the bus is already at full capacity, it does not stop at any more bus stops Time step: 3 s (20 iterations = 1 min) Period modeled: 20 days	Scenario 1: Business as usual (no exclusive bus lane or ancillary measures); Scenario 2: Exclusive bus lane introduced; Scenario 3: Exclusive bus lane + off-board ticketing introduced; Scenario 4: Exclusive bus lane + express bus stops introduced; Scenario 5: Exclusive bus lane + more frequent buses introduced; Scenario 6: All measures introduced	Bus share, travel time, bus travel time, car travel time, length of rush hour	Addition of exclusive bus lane increased bus mode share and reduced travel time for bus and car users; addition of pre-boarding ticket machines is the most effective means of increasing bus modal share and reducing journey times, followed by introduction of higher bus frequencies
33	Okushima and Akiyama (2011)	ABM	Public transport policies	Stylized Tokushima, Japan	Agents: Individuals Interactions: Agent-agent and agent-environment Main processes: Agents choose the commuting mode (car vs. public transport) based on the level of service of each mode and the agents' eco-consciousness; level of service of each mode is defined by travel time (calculated based on traffic flow and capacity) and travel cost; eco-consciousness consists of evaluating the traffic environment in terms of satisfaction with commuting mode and importance of reduction of CO_2_ emissions for the agent; eco-consciousness is also influenced by a strongly-linked agent (friend) such that it is improved if the friend has higher eco-consciousness Time step: 1 week Period modeled: 10 years	Type 1 (1 scenario): Business as usual; Type 2 (5 scenarios): improvement of public transport service + discount of bus fare; Type 3 (6 scenarios): Promotion of individual eco-consciousness by population-wide environmental education; Type 4 (5 scenarios): Strengthening the interaction (cooperation) between individuals	Share of public transport	Improvement of public transport service combined with discount of bus fare achieved the highest impacts in modal shift to bus, followed by strengthening the interaction between individuals
34	Okushima (2015)	ABM	BRT	Stylized Tokushima, Japan	Agents: Individuals Interactions: Agent-agent only Main processes: Agents decision process consists of vehicle purchase and modal shift (car vs. sustainable transport modes); these processes are based on environmental concern (fuel efficiency), mode share in its social network (social conformity), and commuting travel time and cost; vehicle type purchased, and commuting mode chosen feedback to decisions made by other agents in the social network Time step: 1 week Period modeled: 10 years	Type 1 (1 scenario): Business as usual; Type 2 (1 scenario): Implementation of BRT system; Type 3 (3 scenarios): economic incentive policies (cordon pricing, distance tolls, and green tax)	Share of sustainable transport modes	Economic incentive policies maximize the share of sustainable transport modes; BRT implementation resulted in marginal benefits
35	Okushima (2016)	ABM	Active travel promotion	Stylized Tokushima, Japan	Agents: Individuals Interactions: Agent-agent only Main processes: Agents choose the commuting mode (car vs. active travel – walking or cycling) based on health-consciousness, difficulty in modal shift, and trip distance; health-consciousness is influenced by social network such that it is improved if friends have higher health-consciousness Time step: 1 week Period modeled: 10 years	Scenario 1: Business as usual; Scenario 2: population-wide health education (health concerns assumed as factor for modal shift); Scenario 3: promotion of high-performance bicycles; Scenario 4: population-wide health education + promotion high-performance bicycles	Active travel	Interventions did not result in increased active travel, with similar results across scenarios
36	Yang and Diez-Roux (2013)	ABM	Walking promotion	Stylized setting (generic city of 64 km^2^)	Agents: Households, children, schools Interactions: Agent-agent and agent-environment Main processes: Children choose travel to school by walking or are driven by their parents based on distance to school, traffic safety and children's attitude towards walking; each day, traffic safety for each area is updated as a function of the total number of people who walk by that area, and attitude towards walking is updated as a function of the total number of children who walk to school Time step: 1 day Period modeled: 5–10 days	Type 1 (1 scenario): School location evenly distributed and children attend nearest school; Type 2 (3 scenarios): Changing schools' location and catchment area; Type 3 (16 scenarios): Changing schools' size and population density; Type 4 (9 scenarios): Changing traffic safety levels across the city	Walking trips to school	School locations should be evenly distributed over space and children should be assigned to the closest school to maximize the number of children who walk to school; beneficial impacts of smaller catchment areas and higher population density observed; to improve traffic safety, targeting a smaller area around the school with greater intensity seemed to be more effective
37	Yang et al. (2015)	ABM	Public transport policies + walking promotion	Stylized setting (generic city of 64 km^2^)	Agents: Individuals (nested in households) and non-residential locations Interactions: Agent-agent and agent-environment Main processes: Every day, agents choose the travel mode (private automobile, public transportation, or by walking) to non-residential locations based on the attitude towards each mode modified by the cost of each mode; attitude is influenced by traffic congestion and traffic safety: more people driving along the route decreases a person's attitude towards driving and higher level of safety increases a person's attitude towards walking; attitude is also influenced by travel mode of the agents' friends and family members Time step: 1 day Period modeled: 100 days	Type 1 (5 scenarios): Different types of segregation (by income, safety level and land use); Type 3 (4 scenarios): Implementation of transportation cost policies (public transit fares, fuel price, and parking fee); Type 4 (3 scenarios): Policies aimed at changing attitudes towards driving and cycling + transportation cost policies	Percentage of walking trips	Segregation of land use and relative concentration of mixed land uses are important determinants of income differences in walking; safety and income segregation on their own do not have large influences on income differences in walking; very strong disincentives placed on driving and a few incentives offered for taking the bus had the potential for greatly increasing walking trips, particularly at lower income levels
38	Zellner et al. (2016)	ABM	Public transport policies + active travel promotion	Stylized four neighborhoods in the Chicago Metropolitan Region, USA	Agents: Individuals Interactions: Agent-environment only Main processes: There are two types of commuters: those going to downtown (Loop commuters) and those going to other places; the latter are randomly assigned a mode of travel based on current mode share and do not change their travel mode during a simulation; Loop commuters choose a mode (walk to train, bike to destination, bike to train, bus to destination, drive to destination, and shuttle to train) based on the probabilities computed from a utility function comprising monetary cost, time, and perceived safety; perception of safety is updated based on the observed presence of other pedestrians and cyclists in the previous day Time step: 1 h Period modeled: 10 days	Type 1 (1 scenario): Business as usual; Type 2 (1 scenario): Streetscape improvements; Type 3 (1 scenario): Transportation cost policies (public transit fares, fuel price, parking fees); Type 4 (4 scenarios): Provision of shuttles to the neighborhood transit station; Type 5 (1 scenario): Ideal streetscape improvements + ideal transportation cost policy + Ideal provision of shuttles to the neighborhood transit station; Type 6 (21 scenarios): Same as type 5 but modifying distances from transit stations	Mode share	Shuttles have a significant impact and may be robust policies in low-density neighborhoods that have few transportation alternatives to downtown areas or poor coverage of bus service to train station; in areas already offering a good coverage and reliable provision of bus service to train stations; shifts were reinforced by streetscape improvements targeted to areas close to shuttle stops; policies that increase the cost of driving can reinforce the benefits of improving the provision of public transportation
39	Zou et al. (2016)	ABM	Public transport policies	Second ring road of Beijing, China	Agents: Individuals Interactions: Agent-environment only Main processes: Individuals face two types of pre-trip choices: mode selection (car vs. bus/metro) and departure time selection; both choices are influenced by costs, travel time, and level of congestion on the road network; individuals accumulate experience about the performance of the road network then decide whether to stick to the current travel mode and departure time or switch to new ones; if the agent finds a better travel mode and/or departure time, it chooses the new options; the distribution of the travel modes affect the level of congestion on the road network and, consequently, mode speed and travel time Time step: unclear Period modeled: unclear	Type 1 (1 scenario): Business as usual; Type 2 (1 scenario): Increasing demand (number of travelers; Type 3 (7 scenarios): Increasing demand + congestion fees	Modal shift	Travelers shift travel mode and departure time to make the situation better when demand increases, but effect is not significant because the mode changing rate is not so high; congestion charge can increase the changing rate to a large extent

ABM: agent-based modelling; BRT: bus rapid transit.

Five studies tested the impacts of expanding, improving (e.g., creating exclusive bus lanes or changing the BRT system), or incentivizing public transportation formed the focus of five studies (McDonnell and Zellner, 2011; Okushima and Akiyama, 2011; Okushima, 2015; Lemoine et al., 2016a; Zou et al., 2016), three of which were dedicated to BRTs (McDonnell and Zellner, 2011; Okushima, 2015; Lemoine et al., 2016a). Three studies investigated interventions for active travel promotion, such as the creation of cycling infrastructure or implementation of traffic safety measures to promote walking trips to school (Yang and Diez-Roux, 2013; Macmillan et al., 2014; Okushima, 2016). The remaining two studies investigated combinations of public transport and active travel policies, such as the implementation of transportation cost policies (e.g., public transit fares and parking fees), interventions aimed at changing attitudes towards driving and cycling, and streetscape improvements (Yang et al., 2015; Zellner et al., 2016).

Of the 10 simulation studies, four included active travel as the only health-related outcome (Yang and Diez-Roux, 2013; Yang et al., 2015; Lemoine et al., 2016a; Okushima, 2016). One study also analyzed road traffic injuries and all-cause mortality (Macmillan et al., 2014). The other five studies (McDonnell and Zellner, 2011; Okushima and Akiyama, 2011; Okushima, 2015; Zellner et al., 2016; Zou et al., 2016) reported only on transport-related outcomes, such as mode share, mode-specific travel time or mode shift, featuring either bus and/or BRTs as one of the investigated modes.

Nine studies (McDonnell and Zellner, 2011; Okushima and Akiyama, 2011; Yang and Diez-Roux, 2013; Okushima 2015, 2016; Yang et al., 2015; Lemoine et al., 2016a; Zellner et al., 2016; Zou et al., 2016) used ABM, and one (Macmillan et al., 2014) used the system dynamics framework. Only two studies modeled cities in LMIC, specifically, Bogota, Colombia (Lemoine et al., 2016a), and Beijing, China (Zou et al., 2016), whereas three were based on highly stylized settings (i.e., not grounded in any real location) (McDonnell and Zellner, 2011; Yang and Diez-Roux, 2013; Yang et al., 2015). All studies explored both the mechanisms driving the behavior of the investigated systems and tested the potential effects of policy interventions.

Of the 10 simulation studies, five included both agent-agent (or, equivalently, persons-persons in the system dynamics model) and agent-environment (persons-environment) interactions in their models (McDonnell and Zellner, 2011; Okushima and Akiyama, 2011; Yang and Diez-Roux, 2013; Macmillan et al., 2014; Yang et al., 2015). Three modeled agent-environment interactions only (Lemoine et al., 2016a; Zellner et al., 2016; Zou et al., 2016) and the remaining two included just agent-agent interactions (Okushima 2015, 2016). In all studies, policy alternatives were considered as new scenarios through the manipulation of exogenous variables (i.e., there were no agents in the model that enacted policy decisions based on changes in the model).

Of the nine ABM studies, eight (McDonnell and Zellner, 2011; Okushima and Akiyama, 2011; Yang and Diez-Roux, 2013; Okushima 2015, 2016; Yang et al., 2015; Lemoine et al., 2016a; Zellner et al., 2016) applied utility functions to represent the agents' decision-making process and one used production (‘if-then’) rules (Zou et al., 2016). Decisions between modes of transportation were informed by a variety of factors, but most frequently by distance, financial costs and travel time, mode chosen by other persons (such as members of one's friendship network, or those of the community at large), as well as traffic congestion and safety. Mode chosen by other persons, traffic congestion (and, consequently, travel time), and traffic safety were the main mechanisms of interaction between people and with their environment.

Time steps varied significantly across studies, ranging from just three seconds in one study to one year in another. Comparatively, there was less variation in the periods modeled, with four studies (McDonnell and Zellner, 2011; Yang and Diez-Roux, 2013; Lemoine et al., 2016a; Zellner et al., 2016) modelling one month or less, and four other studies modelling 10 years or more (Okushima and Akiyama, 2011; Macmillan et al., 2014; Okushima 2015, 2016).

Five of the 10 studies explored economic incentives to encourage the uptake of mass transit and/or discourage the use of cars (Okushima and Akiyama, 2011; Okushima, 2015; Yang et al., 2015; Zellner et al., 2016; Zou et al., 2016). Evaluated strategies included implementation of congestion fees and green taxes, changes in fuel price and parking fees, and bus fare discounts. The effects of such policies were found to provide significant improvements in health-related outcomes. Improvements of public transport services, including the implementation of BRT systems, were also investigated by five studies (McDonnell and Zellner, 2011; Okushima and Akiyama, 2011; Okushima, 2015; Lemoine et al., 2016a; Zellner et al., 2016). Besides the implementation of BRT lanes, other interventions included increasing bus frequency, the introduction of express bus stops, and changes in the system density (coverage). Overall, these studies showed improvements in active travel time and bus share.

Models demonstrated that sizeable modal shift and changes in health outcomes can be achieved from the implementation of multiple, synergistic policies. For instance, Yang et al., (2015) observed that combinations of decreasing attitudes towards driving, increasing attitudes toward walking, and economic interventions that encourage walking were more effective than any single intervention , in increasing walking for all income levels. Similarly, McDonnell and Zellner, (2011) observed a 14 percentage point increase (21%–35%) in bus share with the implementation of a BRT system, with the potential of reaching a 21 percentage point increase (i.e., 42% of the mode share) with the additional introduction of off-board ticketing.

### 3.3 Quality appraisal

#### 3.3.1 Empirical studies

The studies included in the review employed a range of sampling strategies including, attempts to sample the entire population within a given study area (n = 11) (Chen et al., 2012; Goodman et al., 2013; Goodman et al., 2013b; Dill et al., 2014; Goodman et al., 2014; Panter and Ogilvie, 2015; Bhatia et al., 2016; Brown et al., 2016; Ferenchak and Marshall, 2016; Panter and Ogilvie, 2017; Song et al., 2017); random sampling (n = 4) (Evenson et al., 2005; Cerdá et al., 2012; Pazin et al., 2016; Chang et al., 2017); non-random or stratified sampling (n = 6) (Burbidge and Goulias, 2009; Parker et al. 2011, 2013; Langdon, 2015; Rissel et al., 2015; Panter et al., 2016); purposive sampling (n = 4) (Greaves et al., 2015; Heinen et al., 2015; Heesch et al., 2016; Heinen and Ogilvie, 2016); traffic counts (n = 2) (Boarnet et al., 2005; Cook et al., 2016). The remaining studies (Jensen, 2008; Brown et al., 2016b) provided no description of the sampling strategy used. The representativeness of the recruited participants, as determined by the participant response rate and the sampling strategy used, was unclear for most studies (69%) (Evenson et al., 2005; Jensen, 2008; Parker et al., 2011; Goodman et al., 2013b; Dill et al., 2014; Goodman et al., 2014; Greaves et al., 2015; Heinen et al., 2015; Langdon, 2015; Panter and Ogilvie, 2015; Rissel et al., 2015; Brown et al., 2016; Cook et al., 2016; Heesch et al., 2016; Heinen and Ogilvie, 2016; Panter et al., 2016; Brown et al., 2016b; Chang et al., 2017; Panter and Ogilvie, 2017; Song et al., 2017), only one study was deemed to have a truly representative sample (Goodman et al., 2013), while the rest were somewhat representative (n = 7) (Burbidge and Goulias, 2009; Cerdá et al., 2012; Chen et al., 2012; Parker et al., 2013; Bhatia et al., 2016; Ferenchak and Marshall, 2016; Pazin et al., 2016) or not very representative (n = 1) (Boarnet et al., 2005).

Most studies in the review did not include a control or a comparison group (n = 14) (Boarnet et al., 2005; Evenson et al., 2005; Burbidge and Goulias, 2009; Parker et al., 2011; Goodman et al. 2013b, 2014; Heinen et al., 2015; Langdon, 2015; Bhatia et al., 2016; Cook et al., 2016; Ferenchak and Marshall, 2016; Heinen and Ogilvie, 2016; Panter et al., 2016; Chang et al., 2017), of those that did, the extent to which the control and intervention groups were similar at baseline was unclear for eight studies (Jensen, 2008; Parker et al., 2013; Greaves et al., 2015; Panter and Ogilvie, 2015; Brown et al., 2016; Brown et al., 2016b; Panter and Ogilvie, 2017; Song et al., 2017), while the rest featured control and intervention groups that were comparable at baseline, providing either a quantitative characterization of the two groups (n = 5) (Cerdá et al., 2012; Chen et al., 2012; Dill et al., 2014; Heesch et al., 2016; Pazin et al., 2016) or general statement to that effect (n = 2) (Goodman et al., 2013; Rissel et al., 2015). Included studies featured more self-reported (n = 13) (Evenson et al., 2005; Burbidge and Goulias, 2009; Goodman et al., 2013; Goodman et al. 2013b; Goodman et al. 2014; Heinen et al., 2015; Panter and Ogilvie, 2015; Heinen and Ogilvie, 2016; Panter et al., 2016; Pazin et al., 2016; Chang et al., 2017; Panter and Ogilvie, 2017; Song et al., 2017) than objectively measured outcomes (n = 7) (Boarnet et al., 2005; Jensen, 2008; Parker et al. 2011, 2013; Cerdá et al., 2012; Dill et al., 2014; Brown et al., 2016). There were also studies that reported on both self-reported and objective outcomes (n = 9) (Chen et al., 2012; Greaves et al., 2015; Langdon, 2015; Rissel et al., 2015; Bhatia et al., 2016; Cook et al., 2016; Ferenchak and Marshall, 2016; Heesch et al., 2016; Brown et al., 2016b).

The timing and duration of pre-versus post-intervention assessments varied substantively across studies. Pre-intervention assessments were commonly collected over a period spanning an average of 22 months and ranging from three months to five years. On average, these assessments were conducted 14 months before the intervention and ranged from immediately before to eight years pre-intervention. Post-intervention assessments were collected on average over a period spanning 17 months and ranging from five months to two and a half years. Intervention effects on outcomes were assessed on average eight and a half months after the intervention though some studies conducted post-intervention assessments immediately after, while others conducted their first assessments four years after.

Of the 29 empirical studies, 17 studies experienced loss to follow-up to differing degrees: < 30% attrition was reported by two studies (Cerdá et al., 2012; Dill et al., 2014), 30–59% by 13 studies (Evenson et al., 2005; Goodman et al., 2013b; Goodman et al., 2014; Greaves et al., 2015; Heinen et al., 2015; Panter and Ogilvie, 2015; Rissel et al., 2015; Brown et al., 2016; Heinen and Ogilvie, 2016; Panter et al., 2016; Pazin et al., 2016; Brown et al., 2016b; Song et al., 2017), and 60–89% by two studies (Burbidge and Goulias, 2009; Panter and Ogilvie, 2017). Please refer to Appendix 4 for more information about the quality appraisal of included studies.

#### 3.3.2 Simulation studies

Overall, the system-based simulation studies made explicit the models’ assumptions and structure. Nine studies provided justifications for all or most of their assumptions (McDonnell and Zellner, 2011; Okushima and Akiyama, 2011; Yang and Diez-Roux, 2013; Macmillan et al., 2014; Okushima 2015, 2016; Yang et al., 2015; Zellner et al., 2016; Zou et al., 2016). Only one study did not provide justifications for the equations used (McDonnell and Zellner, 2011) while the four other studies (Macmillan et al., 2014; Yang et al., 2015; Lemoine et al., 2016a; Zellner et al., 2016) provided justifications only for some of the equations. All 10 system-based simulation studies used empirical sources to inform their parameters.

Calibration and validation procedures and sensitivity or uncertainty analysis were infrequently assessed. Among the four studies with some parameters that could not be informed by empirical data (McDonnell and Zellner, 2011; Okushima and Akiyama, 2011; Yang and Diez-Roux, 2013; Zou et al., 2016), two (McDonnell and Zellner, 2011; Okushima and Akiyama, 2011) did not calibrate the unknown parameter values and the other two (Yang and Diez-Roux, 2013; Zou et al., 2016) used categorical calibration only (i.e., searched for parameter values that produce model results within a range acceptably close to data). Six studies (Okushima and Akiyama, 2011; Yang and Diez-Roux, 2013; Okushima 2015, 2016; Zellner et al., 2016; Zou et al., 2016) did not validate the results of baseline scenarios against expected (in purely stylized models) or observed (in models grounded on real locations) outcomes, and only two studies (McDonnell and Zellner, 2011; Macmillan et al., 2014) compared outcomes using either qualitative or quantitative means. Five studies (Yang and Diez-Roux, 2013; Okushima 2015, 2016; Yang et al., 2015; Zou et al., 2016) did not conduct either sensitivity or uncertainty analyses, only one conducted both (Macmillan et al., 2014), and the remaining four (McDonnell and Zellner, 2011; Okushima and Akiyama, 2011; Lemoine et al., 2016a; Zellner et al., 2016) ran sensitivity analyses only. Appendix 5 provides more information about the quality appraisal of included simulation studies.

## 4 Discussion

Of the 39 empirical and system-based simulation studies identified, the majority focused on bicycle lanes and BRT systems. Notably, only one study evaluated aerial trams, and none investigated Open Streets programs. Moreover, most studies (n = 24) focused on HIC, while only five studies explored cities in LMIC. In *empirical studies*, bicycle lane interventions were associated with increases in physical activity and active transport. Similarly, BRT systems with an adjacent bicycle lane were found to promote active travel and walking for transport and recreation. The sole aerial tram study reported a significant decrease in homicides following the aerial tram installation. There was also some evidence to suggest that multiple component interventions may be more effective than single component interventions in increasing physical activity. *System-based simulation studies* showed that economic incentives designed to disincentivise car use, and policies designed to improve the public transportation system, can have positive impacts on active travel time and bus share. Consistent with empirical studies evaluating multicomponent interventions, systems-based simulations often reported synergistic effects of multiple interventions and policies.

The *quality of included studies* was mixed. Most empirical studies did not include a control or comparison group and for those that did, it was largely unclear to what extent the control and intervention groups were similar at baseline. Empirical studies also reported a range of sampling approaches. There existed substantive uncertainty about the generalizability of study findings because 21 of the 29 included studies featured populations that were either not representative of the study region or whose representativeness was unclear. System-based simulation studies commonly provided justifications for the assumptions made and the equations used, although several were highly abstract models. Most simulation studies were also informed by empirical data. However, calibration and validation procedures and uncertainty analysis were infrequently conducted, and the periods modeled in a handful of studies appeared relatively short (less than 1 month), which may have impacted study findings.

The most common policy evaluated by empirical studies pertained to *bicycle infrastructure*. These studies generally reported beneficial effects on bicycle mode share and active transport duration and number of trips. Some adverse effects on injuries were documented although these were reported by a single study. However, only one of these longitudinal studies was based in or explored cities in LMIC. Prior reviews have also identified research on bicycle infrastructure in LMIC as important gaps (Fraser and Lock, 2010; Yang et al., 2010). Cross-sectional studies focused on LMIC report consistent effects of bicycle lanes on active travel. For example, Florindo et al. found that people living within 500 meters of bicycle paths in Sao Paolo, Brazil were more than twice more likely to engage in cycling for transportation than those who lived further away (Florindo et al., 2018). Another study based in Taiwan, found an association between residents’ perceptions of bicycle lanes in their neighborhood and past week cycling for transportation among adults (Liao et al., 2015). Additional longitudinal evaluation studies are needed to determine the impact of the rapid growth in bicycle infrastructures that is occurring in many LMIC, in contexts that are very different from those of HIC.

The second most explored policy focused on *BRT systems*. These studies, mostly focused on HIC, found that BRT systems increase active travel and walking for transport and recreation. There are however over 160 cities around the world with operating BRT systems (BRT+ Centre of Excellence and EMBARQ, 2019). Among them are cities from a range of LMIC in Latin America (e.g., Brazil, Colombia, Argentina, Mexico), Asia (e.g., China, India, Taiwan, Vietnam) and the African continent (e.g., Morocco, South Africa, Nigeria, Uganda). While few longitudinal studies have investigated these transport systems in LMIC, cross-sectional analyses suggest that BRT use may positively impact health outcomes. For example, using cross-sectional survey data, Lemoine et al., 2016b found that BRT use in Bogota, Colombia, was associated with around 12 min of moderate-to-vigorous physical activity each day (Lemoine et al., 2016b). Similarly, Bartels et al. used cross-sectional intercept surveys of BRT passengers in Cape Town, South Africa, and found that BRT-users engaged in significantly longer periods of physical activity per week and were over twice more likely to achieve recommended physical activity guidelines than non-users (Bartels et al., 2016). These findings align closely with the general findings of our review which suggest that BRT interventions represent effective means for increasing physical activity and active travel.

*Aerial trams* form part of the transit infrastructure, both in LMIC and HIC around the world (Alshalalfah et al., 2012). However, our review of the literature indicates that aerial trams remain relatively under-studied, particularly with respect to their health-related impacts. We found only one longitudinal evaluation of aerial trams and no system-based simulation studies. The sole study included in our review, which capitalized on a natural experiment, found that the construction of the Metrocable (i.e., aerial tram) in Medellin, Colombia, resulted in significant reductions in homicide rates in the neighborhoods surrounding the new aerial tram (Cerdá et al., 2012). Other studies which did not meet the inclusion criteria of our review investigated the impacts of aerial trams on relatively distal outcomes indirectly related to health and health-related behavior, for example, employment access and travel time. One of these studies found that the Metrocable significantly improved access to the central business district and thereby more than doubled employment opportunities for aerial tram users, including low-income groups (Bocarejo et al., 2014). Moreover, in their study of the Mi Teleférico aerial tram in La Paz, Bolivia, Garsous et al., (2019) found that users of the aerial tram reduced their travel time by around 20%. Travel time reductions such as these have variously been linked to improved access to health care, employment and education as well as opportunities for leisure-time activities including physical activity (Garsous et al., 2019).

Despite the increasing prominence of *Open Streets programs* in both LMIC and HIC, our review did not identify any longitudinal evaluations of the health impacts of these initiatives. Sarmiento et al. identified 38 Open Streets programs implemented across 11 different countries in 2010, most of which were concentrated in Latin America (Sarmiento et al., 2010). Another review identified Open Streets initiatives hosted in 47 different US cities (Kuhlberg et al., 2014). Furthermore, cross-sectional evidence suggests that Open Streets initiatives can confer meaningful public health benefits. For example, using information synthesized from the Open Streets programs, Sarmiento et al. estimated important potential contributions of Ciclovias to physical activity (Sarmiento et al., 2010). Positive associations have also been observed among school children. For example, Triana et al. found that frequent Ciclovia use among Colombian school children was associated with significantly lower levels of sedentary time and higher moderate-to-vigorous physical activity on Sundays, but interestingly, not weekdays (Triana et al., 2019). However, longitudinal evaluations of these important initiatives are necessary.

We observed several points of alignment between the *system-based simulation papers and* the *longitudinal evaluation studies* included in our review. For example, both empirical and simulation studies focused predominantly on estimating the impact of policies encompassing expansions and or improvements in public transport infrastructure (e.g., express bus lanes, creation of BRT system) and service delivery (e.g., increased frequency of buses), or incentives for public transportation (e.g., fare changes) on health. Evidence across both bodies of literature suggests that multi-pronged interventions may be more effective than single-component interventions in shaping some health outcomes. This observation is in keeping with published research (van Sluijs et al., 2007) and Social Ecological Theory which posits that ecological approaches, which seek to enact change at multiple levels of a system, are more effective than those targeted toward just one level (Green et al., 1996; Sallis et al., 2008). However, it was unclear from our systematic review which of the intervention components in a given multi-component study were the drivers of the observed health impacts.

Another important observation was that all simulation studies captured by our review used empirical sources to inform the selection and characterization of model parameters, highlighting the importance of robust empirical studies focused on exploring the influence of transportation policies on health outcomes across a range of contexts, not just HIC. This reliance on empirical data and the fact that most empirical studies we identified were conducted in HIC could explain why most of the simulation-based papers included in our review also explored high-income contexts.

We observed heterogeneity in how studies operationalized people's *exposure to a given transportation intervention*. Some studies compared geographic locations while others considered intervention impacts among people living in an area of intervention influence. These areas were either defined using existing geographic units (e.g., census blocks) or buffers centred on the focal point of an intervention. Only a subset of empirical studies compared the health effects of a given intervention for those living different distances from the intervention site, despite contemporary debates advocating for a pluralistic measurement approach (Laatikainen et al., 2018). Most of these studies reported more significant and positive effects on health outcomes for those living closer to the site of an intervention than those living further away; a finding consistent with existing research (McCormack and Shiell, 2011; Djurhuus et al., 2014).

The studies in our review assessed a wide *range of health outcomes*, however, anthropometric/physiological measures, and mortality outcomes were infrequently reported. Moreover, none of the studies in our review considered disease outcomes such as diabetes, or respiratory and mental health outcomes. And, strikingly, few studies addressed issues of equity by exploring intervention effects using stratified analyses which would have enabled critical insights into health inequalities by socioeconomic status and demographic factors such as race and gender. This relative underrepresentation of *equity-grounded research* has been observed in other reviews of transportation systems in LMIC (Yanez-Pagans et al., 2018) and those investigating built environment influences on physical activity and active transport more broadly (Smith et al., 2017).

The accessibility of transportation is an important predictor of health care access (Syed et al., 2013), and employment, which has been linked to a range of health-related behaviors through its influence on both income and time scarcity (Venn and Strazdins, 2017). Importantly, research based in Latin America has shown that patterns in access to BRT, by income, can vary from one city to another. For example, the BRT system in Lima, Peru has been shown to predominantly benefit middle- and higher-SES groups due to the systems limited coverage of areas with high concentrations of poor residents (Oviedo et al., 2019). On the other hand, in Cali, Colombia, the highest levels of access to a new BRT system were observed among middle income groups, while residents of predominantly low and high income neighborhoods had far more limited access (Delmelle and Casas, 2012). Such inequalities however are not only observed in LMIC, they have also been reported in HIC such as Australia (Ricciardi et al., 2015), with observed inequalities in access spanning both age and the socioeconomic spectrum. Thus, additional evaluations on the impact of these types of interventions on equity outcomes is sorely needed.

### 4.1 Limitations

This review should be considered with a few limitations in mind. Our review was necessarily limited through the exclusion of papers that assessed the impacts of BRT, aerial trams, bicycle lanes and Open Streets programs on outcomes that have implications for health, such as accidents, crashes and traffic-related air pollution, but that are not themselves health outcomes. We also excluded studies employing non-longitudinal study designs and other simulation methods such as health impact assessment models, social network analysis, microsimulation or more conceptual/qualitative models arising from participatory approaches such as group model building. To focus the scope of our review, we also excluded studies evaluating light rail transit systems, as well as those focused specifically on bicycle boxes, intersection crossings or roundabouts as opposed to continuous street segments. Given time and resource constraints we did not contact study authors for clarification where information was missing or unclear. Most of the studies we included in the review were based in HIC which may limit the generalizability of our findings to LMIC. Our assessment of the quality of system-based simulation studies may also be limited given the lack of guidance on how to assess the quality of these types of studies. Finally, several papers included in the review evaluated the same intervention. For example, five different papers, all conducted as part of the iConnect Study, evaluated the same set of bicycle lane interventions implemented in three cities in the United Kingdom (UK) (Goodman et al. 2013b, 2014; Panter and Ogilvie 2015, 2017; Song et al., 2017), while four papers evaluated the same combined BRT and bicycle lane intervention in Cambridge, UK (Heinen et al., 2015; Heinen and Ogilvie, 2016; Panter et al., 2016; Chang et al., 2017). Given this overlap, our findings represent outcomes for just 19 unique interventions variously evaluated by the 29 empirical studies included in the review.

### 4.2 Recommendations for future research

The findings of our review highlight several important areas for future research. First, more evaluation and system-based simulation studies are needed to assess the influence of bicycle lanes, BRT systems, aerial trams and Open Streets programs on health outcomes, particularly in LMIC. This is particularly important as differences between HIC and LMIC have been observed in studies investigating associations between built environment characteristics and physical activity, for example (Cleland et al., 2019). In the case of empirical studies, rigorous designs including representative population samples, valid comparison groups, and before and after assessments are needed. On the other hand, the use of evidence to justify model rules and parameters is critical for simulation studies. Second, there also exists a need for studies replicating policy evaluations in different cities to determine the extent to which city-level factors impact the effectiveness of interventions overall and for different population subgroups. These studies may in turn inform simulation-based studies which have the capacity to identify under what conditions a given policy or combinations of policies may be most effective in promoting health outcomes across the socioeconomic and demographic spectrum. Systems-based simulation methods can be especially useful as policy decision-tools, particularly in LMIC where the assessment of large-scale population-level interventions may be prohibitively expensive or impractical to test in the real world (Hammond, 2015). These methods can also raise new questions and in turn inform the focus of empirical research and evaluation studies (Diez Roux, 2019). To support the use of system-based simulation methods, tools guiding the assessment of quality for these studies represents an important area for future research. Participatory processes such as group model building (Hovmand, 2014) which seek to elicit the perspectives of diverse stakeholders, can play an important role in informing the design of simulation models. Moreover, they have the potential to elucidate novel evaluation targets and foster intersectoral and community partnerships which can play an important role in the sustainability and longevity of interventions.

Third, prospective studies, both empirical and simulation-based, focusing on BRT, bicycle lanes, aerial trams and Open Streets programs ought to explore the potential environmental and health co-benefits of these policies. The high expansion of new programs in both HIC and LMIC provide a unique opportunity for natural experiments. For example, studies investigating bicycle lane interventions would be well placed to investigate changes in transport-related air pollution and the respiratory health of city residents alongside and in interaction with changes in mode share and physical activity during transport and leisure time. More studies evaluating the influence of bicycle lanes on injury outcomes are also required. Fourth, studies employing stratified analysis, by for example, gender, income, age and race are required to explore the impact of these transport policies on health disparities across a range of outcomes including anthropometric and physiological measures as well as respiratory and other disease outcomes. Relatedly, to ensure study quality, the design of future system-based simulation studies ought to reflect an alignment between the outcomes of interest and the timeframes being modeled.

Finally, researchers seeking to advance research in this area, particularly in LMIC may benefit from the use of innovative and relatively cost-effective data collection methods such as street imagery (e.g., Google Street View and Bing StreetSide) to capture granular information about the physical environment and behavioral data. Street imagery has widely been tested as a built environment audit tool, including its predictive capacity in documenting relatively small-scale historic changes to the built environment (Candido et al., 2018). There is also evidence to suggest that it can be used to estimate pedestrian counts (Yin et al., 2015), and city-level travel patterns including census-reported mode share (i.e., walking and public transit use, cycling, motorcycle and car use) as well as survey-reported past-month participation in cycling (Goel et al., 2018). Given these features, and with years of historical data available, street imagery may provide an avenue for the conduct of retrospective longitudinal policy evaluations of transport policies, and a promising means of complementing traditional data collection methods, particularly in LMIC.

Researchers can also benefit from using a citizen science approach which “empowers residents to collect diagnostic information about their community environment, prioritize areas of concern, and engage in cross-sector collaboration to generate practical and impactful solutions” (King et al., 2016, p.31). These approaches have successfully been used in Latin American countries to collect neighborhood-level information as well as qualitative data relating to a range of initiatives, including Open Streets programs (King et al., 2016). Other emerging approaches that might be leveraged to advance future studies seeking to monitor the impact of transport policies include drone technology, which has been used to collect data on pedestrian counts (Park and Ewing) and deep learning image analysis, which may afford an especially promising and cost-effective means of estimating local environmental exposures and spatial inequalities in income, education, employment and health, particularly in LMIC (Suel et al., 2019; Weichenthal et al., 2019).

## 5 Conclusion

The literature base encompassing longitudinal evaluations, and system-based simulation studies exploring the health impacts of BRT systems, bicycle lane and aerial tram infrastructure and Open Streets programs varies widely by transportation policy and geographical context. This review contributes to the literature by highlighting several important gaps in knowledge. Specifically, it highlights an underrepresentation of certain types of transportation policies (i.e., aerial trams and Open Streets programs), outcomes (e.g., physiological, anthropometric and health equity measures), and countries (i.e, LMIC) within the literature. By synthesizing the available research, this review also identifies bike lanes and BRT systems as promising transportation initiatives for promoting physical activity and active travel at the population-level. Finally, it provides a series of recommendations for future research designed to bridge critical gaps in understanding, and to support the advancement of the public health agenda through transportation policy.

## Author contributions

IS initiated and conceptualised the review, IS and LMTG developed the search strategy, the extraction and reporting tools and screened studies for inclusion with the help of NG and BH. IS, LMTG, MAM, FM and JDM performed duplicate extractions and assessment of quality. IS and LMTG synthesized the studies in the review. IS wrote the first draft of the manuscript and critically revised subsequent versions with writing contributions from LMTG and AVDR. AVDR supervised the project and provided critical guidance throughout. All authors discussed the search strategy, extraction and reporting tools and results, suggested revisions and contributed to the final manuscript.

## Funding sources

The Salud Urbana en América Latina (SALURBAL)/Urban Health in Latin America project is funded by the 10.13039/100010269Wellcome Trust, UK [grant 205177/Z/16/Z]. More information about the project can be found at www.lacurbanhealth.org. We acknowledge the support of SALURBAL investigators. For more information on SALURBAL and to see a full list of investigators see https://drexel.edu/lac/salurbal/team/.

LMTG worked under the auspices of the 10.13039/501100011032Centre for Diet and Activity Research (CEDAR), a UKCRC Public Health Research Centre of Excellence which is funded by the 10.13039/501100000274British Heart Foundation, 10.13039/501100000289Cancer Research UK, 10.13039/501100000269Economic and Social Research Council, 10.13039/501100000265Medical Research Council, the 10.13039/501100000272National Institute for Health Research, UK and the 10.13039/100010269Wellcome Trust [grant MR/K023187/1]. JDM was funded by the Universidad de Ibagué, Colombia [Project 17468 INT]. FM was funded by the 10.13039/100000002National Institutes of Health Fogarty International Center, USA [grant D43TW010540], and by the FAPA grant from the 10.13039/501100006070Universidad de los Andes, Colombia.

## Declaration of competing interests

The authors declare that they have no known competing financial interests or personal relationships that could have appeared to influence the work reported in this paper.

## Footnotes

Appendix A Supplementary data to this article can be found online at https://doi.org/10.1016/j.envres.2020.109519.

## References

[bib1] Alshalalfah B., Shalaby A., Dale S., Othman F.M.Y. (2012). Aerial ropeway transportation systems in the urban environment: state of the art. J. Transport. Eng..

[bib2] Babisch W. (2006). Transportation noise and cardiovascular risk: updated review and synthesis of epidemiological studies indicate that the evidence has increased. Noise Health.

[bib3] Bartels C., Kolbe-Alexander T., Behrens R., Hendricks S., Lambert E.V. (2016). Can the use of Bus Rapid Transit lead to a healthier lifestyle in urban South Africa? The SUN Study.". J. Trans. Health.

[bib4] Becerra J., Reis R., Frank L., Ramirez-Marrero F., Welle B., Arriaga Cordero E., Mendez Paz F., Crespo C., Dujon V., Jacoby E., Dill J., Weigand L., Padin C. (2013). Transport and health: a look at three Latin American cities. Cad. Saúde Pública.

[bib5] Bhatia D., Richmond S.A., Loo C.K.J., Rothman L., Macarthur C., Howard A. (2016). "Examining the impact of cycle lanes on cyclist-motor vehicle collisions in the city of Toronto.". J. Trans. Health.

[bib6] Boarnet M., Day K., Anderson C., McMillan T., Alfonzo M. (2005). California's safe routes to school program: impacts on walking, bicycling, and pedestrian safety. J. Am. Plann. Assoc..

[bib7] Bocarejo J.P., Portilla I.J., Velásquez J.M., Cruz M.N., Peña A., Oviedo D.R. (2014). "An innovative transit system and its impact on low income users: the case of the Metrocable in Medellín.". J. Transport Geogr..

[bib8] Borrell C., Pons-Vigués M., Morrison J., Díez È. (2013). Factors and processes influencing health inequalities in urban areas. Journal of Epidemiology and Community Health.

[bib9] Brown B.B., Smith K.R., Tharp D., Werner C.M., Tribby C.P., Miller H.J., Jensen W. (2016). A complete street intervention for walking to transit, nontransit walking, and bicycling: a quasi-experimental demonstration of increased use.". J. Phys. Activ. Health.

[bib10] Brown B.B., Tharp D., Tribby C.P., Smith K.R., Miller H.J., Werner C.M. (2016). Changes in bicycling over time associated with a new bike lane: relations with kilocalories energy expenditure and body mass index. J. Trans. Health.

[bib11] BRT+ Centre of Excellence and EMBARQ (2019). EMBARQ and centre of excellence for BRT. http://www.brtdata.org.

[bib12] Brunekreef B., Holgate S.T. (2002). "Air pollution and health.". Lancet.

[bib13] Burbidge S.K., Goulias K.G. (2009). "Evaluating the impact of neighborhood trail development on active travel behavior and overall physical activity of suburban residents.". Transport. Res. Rec..

[bib14] Candido R.L., Steinmetz-Wood M., Morency P., Kestens Y. (2018). Reassessing urban health interventions: back to the future with Google street View time machine. Am. J. Prev. Med..

[bib15] Cerdá M., Morenoff J.D., Hansen B.B., Tessari Hicks K.J., Duque L.F., Restrepo A., Diez-Roux A.V. (2012). Reducing violence by transforming neighborhoods: a natural experiment in Medellín, Colombia.". Am. J. Epidemiol..

[bib16] Chang A., Miranda-Moreno L., Cao J., Welle B. (2017). The effect of BRT implementation and streetscape redesign on physical activity: a case study of Mexico City.". Transport. Res. Pol. Pract..

[bib17] Chen L., Chen C., Srinivasan R., McKnight C.E., Ewing R., Roe M. (2012). "Evaluating the safety effects of bicycle lanes in New York City.". Am. J. Pub. Health.

[bib18] Cleland C., Reis R.S., Ferreira Hino A.A., Hunter R., Fermino R.C., Koller de Paiva H., Czestschuk B., Ellis G. (2019). Built environment correlates of physical activity and sedentary behaviour in older adults: a comparative review between high and low-middle income countries.". Health Place.

[bib19] Cook T.J., O'Brien S.W., Jackson K.N., Findley D.J., Searcy S.E. (2016). "Behavioral effects of completing a critical link in the american tobacco trail.". Transport. Res. Rec..

[bib20] Covidence (2017). Covidence: A Cochrane Technology Platform.

[bib21] Delmelle E.C., Casas I. (2012). Evaluating the spatial equity of bus rapid transit-based accessibility patterns in a developing country: the case of Cali, Colombia. Transport Pol..

[bib22] Diez Roux A.V. (2019). The unique space of epidemiology: drawing on the past to project into the future. Am. J. Epidemiol.

[bib23] Dill J., McNeil N., Broach J., Ma L. (2014). Bicycle boulevards and changes in physical activity and active transportation: findings from a natural experiment. Prev. Med..

[bib24] Djurhuus S., Hansen H., Aadahl M., Glümer C. (2014). The association between access to public transportation and self-reported active commuting. Int. J. Environ. Res. Publ. Health.

[bib25] Evenson K.R., Herring A.H., Huston S.L. (2005). "Evaluating change in physical activity with the building of a multi-use trail.". Am. J. Prev. Med..

[bib26] Ferenchak N.N., Marshall W.E. (2016). The relative (in)effectiveness of bicycle sharrows on ridership and safety outcomes. Transportation Research Board 95th Annual Meeting. Washington DC, United States, Transportation Research Board: 17.

[bib27] Florindo A.A., Barrozo L.V., Turrell G., Barbosa J., Cabral-Miranda W., Cesar C.L.G., Goldbaum M. (2018). Cycling for transportation in Sao Paulo City: associations with bike paths, train and subway stations.". Int. J. Environ. Res. Publ. Health.

[bib28] Fraser S.D.S., Lock K. (2010). "Cycling for transport and public health: a systematic review of the effect of the environment on cycling.". Eur. J. Publ. Health.

[bib29] Garsous G., Suárez-Alemán A., Serebrisky T. (2019). Cable cars in urban transport: travel time savings from La Paz-El Alto (Bolivia). Transport Pol..

[bib30] Goel R., Garcia L.M., Goodman A., Johnson R., Aldred R., Murugesan M., Brage S., Bhalla K., Woodcock J. (2018). Estimating city-level travel patterns using street imagery: a case study of using Google Street View in Britain.". PloS One.

[bib31] Goodman A., Panter J., Sharp S.J., Ogilvie D. (2013). Effectiveness and equity impacts of town-wide cycling initiatives in England: a longitudinal, controlled natural experimental study.". Soc. Sci. Med..

[bib32] Goodman A., Sahlqvist S., Ogilvie D. (2013). Who uses new walking and cycling infrastructure and how? Longitudinal results from the UK iConnect study. Prev. Med..

[bib33] Goodman A., Sahlqvist S., Ogilvie D. (2014). New walking and cycling routes and increased physical activity: one- and 2-year findings from the UK iConnect Study. Am. J. Pub. Health.

[bib34] Greaves S., Ellison R., Ellison A., Crane M., Rissel C., Standen C. (2015). Changes in Cycling Following an Infrastructure Intervention. 37th Australasian Transportation Research Forum.

[bib35] Green L.W., Richard L., Potvin L. (1996). "Ecological foundations of health promotion.". Am. J. Health Promot..

[bib36] Hammond R.A. (2015). Appendix A Considerations and Best Practices in Agent-Based Modeling to Inform Policy. Assessing the Use of Agent-Based Models for Tobacco Regulation. R. Wallace, A. Geller and V. Ogawa.

[bib37] Heesch K.C., James B., Washington T.L., Zuniga K., Burke M. (2016). Evaluation of the Veloway 1: a natural experiment of new bicycle infrastructure in Brisbane, Australia.". J. Trans. Health.

[bib38] Heinen E., Ogilvie D. (2016). Variability in baseline travel behaviour as a predictor of changes in commuting by active travel, car and public transport: a natural experimental study. J. Trans. Health.

[bib39] Heinen E., Panter J., Mackett R., Ogilvie D. (2015). "Changes in mode of travel to work: a natural experimental study of new transport infrastructure.". Int. J. Behav. Nutr. Phys. Activ..

[bib40] Higgins J., Lasserson T., Chandler J., Tovey D., Churchill R. (2016). Standards for the Conduct of New Cochrane Intervention Reviews. Methodological Expectations of Cochrane Intervention Reviews. J. Higgins, T. Lasserson, J. Chandler, D. Tovey and R. Churchill.

[bib41] Hovmand P. (2014). Group Model Building and Community-Based System Dynamics Process. Community Based System Dynamics.

[bib42] Jayasinghe S. (2011). Conceptualising population health: from mechanistic thinking to complexity science.". Emerg. Themes Epidemiol..

[bib43] Jensen S.U. (2008). Bicycle tracks and lanes: a before-after study.

[bib44] King A.C., Winter S.J., Sheats J.L., Rosas L.G., Buman M.P., Salvo D., Rodriguez N.M., Seguin R.A., Moran M., Garber R., Broderick B., Zieff S.G., Sarmiento O.L., Gonzalez S.A., Banchoff A., Dommarco J.R. (2016). "Leveraging citizen science and information technology for population physical activity promotion.". Trans. J. Am. College Sports Med..

[bib45] Kuhlberg J.A., Hipp J.A., Eyler A., Chang G. (2014). Open streets initiatives in the United States: closed to traffic, open to physical activity. J. Phys. Activ. Health.

[bib46] Laatikainen T.E., Hasanzadeh K., Kyttä M. (2018). "Capturing exposure in environmental health research: challenges and opportunities of different activity space models.". Int. J. Health Geogr..

[bib47] Langdon M. (2015). An Evidence-Based Assessment of the Impact of Cycling Infrastructure in South East Queensland. Australian Institute of Traffic Planning and Management National Conference.

[bib48] Lee I.M., Shiroma E.J., Lobelo F., Puska P., Blair S.N., Katzmarzyk P.T. (2012). Effect of physical inactivity on major non-communicable diseases worldwide: an analysis of burden of disease and life expectancy. Lancet.

[bib49] Lemoine P.D., Cordovez J.M., Zambrano J.M., Sarmiento O.L., Meisel J.D., Valdivia J.A., Zarama R. (2016). "Using agent based modeling to assess the effect of increased Bus Rapid Transit system infrastructure on walking for transportation.". Prev. Med..

[bib50] Lemoine P.D., Sarmiento O.L., Pinzón J.D., Meisel J.D., Montes F., Hidalgo D., Pratt M., Zambrano J.M., Cordovez J.M., Zarama R. (2016). “TransMilenio, a scalable bus rapid transit system for promoting physical activity.”. J. Urban Health.

[bib51] Li Y., Lawley M.A., Siscovick D.S., Zhang D., Pagan J.A. (2016). Agent-based modeling of chronic diseases: a narrative review and future research directions.". Prev. Chronic Dis..

[bib52] Liao Y., Wang I.T., Hsu H.-H., Chang S.-H. (2015). "Perceived environmental and personal factors associated with walking and cycling for transportation in Taiwanese adults.". Int. J. Environ. Res. Publ. Health.

[bib53] Litman T. (2013). "Transportation and public health.". Annu. Rev. Publ. Health.

[bib54] Lucas K. (2012). Transport and social exclusion: where are we now?. Transport Pol..

[bib55] Macmillan A., Connor J., Witten K., Kearns R., Rees D., Woodward A. (2014). The societal costs and benefits of commuter bicycling: simulating the effects of specific policies using system dynamics modeling.". Environ. Health Perspect..

[bib56] McCormack G.R., Shiell A. (2011). "In search of causality: a systematic review of the relationship between the built environment and physical activity among adults.". Int. J. Behav. Nutr. Phys. Activ..

[bib57] McDonnell S., Zellner M. (2011). "Exploring the effectiveness of bus rapid transit a prototype agent-based model of commuting behavior.". Transport Pol..

[bib58] Moher D., Liberati A., Tetzlaff J., Altman D.G., The PRISMA Group (2009). Preferred reporting items for systematic reviews and meta-analyses: the PRISMA statement.". PLoS Med..

[bib59] Nianogo R.A., Arah O.A. (2015). Agent-based modeling of noncommunicable diseases: a systematic review.". Am. J. Pub. Health.

[bib60] Okushima M. (2015). "Simulating social influences on sustainable mobility shifts for heterogeneous agents.". Transportation.

[bib61] Okushima M. (2016). Multi-agent Simulation of Commuting Modal Shift Considering with Health Conscious. 2016 Joint 8th International Conference on Soft Computing and Intelligent Systems and 17th International Symposium on Advanced Intelligent Systems.

[bib62] Okushima M., Akiyama T. (2011). "Multi-agent transport simulation model for eco-commuting promotion planning.". J. Adv. Comput. Intell. Intell. Inf..

[bib63] Oviedo D., Scholl L., Innao M., Pedraza L. (2019). Do bus rapid transit systems improve accessibility to job opportunities for the poor? The case of Lima, Peru.". Sustainability.

[bib64] Panter J., Heinen E., Mackett R., Ogilvie D. (2016). "Impact of new transport infrastructure on walking, cycling, and physical activity.". Am. J. Prev. Med..

[bib65] Panter J., Ogilvie D. (2015). "Theorising and testing environmental pathways to behaviour change: natural experimental study of the perception and use of new infrastructure to promote walking and cycling in local communities.". British Med. J. Open.

[bib66] Panter J., Ogilvie D. (2017). Can environmental improvement change the population distribution of walking?. J. Epidemiol. Community.

[bib67] Park, K. and R. Ewing "The usability of Unmanned Aerial Vehicles (UAVs) for pedestrian observation." J. Plann. Educ. Res. 0(0): 0739456X18805154.

[bib68] Parker K.M., Gustat J., Rice J.C. (2011). Installation of bicycle lanes and increased ridership in an urban, mixed-income setting in New Orleans, Louisiana. J. Phys. Activ. Health.

[bib69] Parker K.M., Rice J., Gustat J., Ruley J., Spriggs A., Johnson C. (2013). "Effect of bike lane infrastructure improvements on ridership in one New Orleans neighborhood.". Ann. Behav. Med..

[bib70] Pazin J., Garcia L.M.T., Florindo A.A., Peres M.A., Guimarães A.C.d.A., Borgatto A.F., Duarte M.d.F.d.S. (2016). Effects of a new walking and cycling route on leisure-time physical activity of Brazilian adults: a longitudinal quasi-experiment.". Health Place.

[bib71] Pucher J., Dill J., Handy S. (2010). Infrastructure, programs, and policies to increase bicycling: an international review. Prev. Med..

[bib72] Ricciardi A.M., Xia J., Currie G. (2015). Exploring public transport equity between separate disadvantaged cohorts: a case study in Perth, Australia. J. Transport Geogr..

[bib73] Rissel C., Greaves S., Wen L.M., Crane M., Standen C. (2015). Use of and short-term impacts of new cycling infrastructure in inner-Sydney, Australia: a quasi-experimental design. Int. J. Behav. Nutr. Phys. Activ..

[bib74] Rydin Y., Bleahu A., Davies M., Dávila J.D., Friel S., De Grandis G., Groce N., Hallal P.C., Hamilton I., Howden-Chapman P., Lai K.-M., Lim C.J., Martins J., Osrin D., Ridley I., Scott I., Taylor M., Wilkinson P., Wilson J. (2012). "Shaping cities for health: complexity and the planning of urban environments in the 21st century.". Lancet.

[bib75] Sallis J.F., Owen N., Fisher E.B., Glanz K., Rimer B.K., Viswanath K. (2008). Ecological Models of Health Behaviour. Health Behavior and Health Education: Theory, Research and Practice.

[bib76] Sarmiento O., Torres A., Jacoby E., Pratt M., Schmid T.L., Stierling G. (2010). The Ciclovia-Recreativa: a mass-recreational program with public health potential.". J. Phys. Activ. Health.

[bib77] Sarmiento O.L., Díaz del Castillo A., Triana C.A., Acevedo M.J., Gonzalez S.A., Pratt M. (2017). Reclaiming the streets for people: insights from ciclovías recreativas in Latin America. Prev. Med..

[bib78] Smith M., Hosking J., Woodward A., Witten K., MacMillan A., Field A., Baas P., Mackie H. (2017). "Systematic literature review of built environment effects on physical activity and active transport – an update and new findings on health equity.". Int. J. Behav. Nutr. Phys. Activ..

[bib79] Song Y., Preston J., Ogilvie D. (2017). New walking and cycling infrastructure and modal shift in the UK: a quasi-experimental panel study." Transportation Research Part A:. Policy and Practice.

[bib80] Suel E., Polak J.W., Bennett J.E., Ezzati M. (2019). "Measuring social, environmental and health inequalities using deep learning and street imagery.". Sci. Rep..

[bib81] Syed S.T., Gerber B.S., Sharp L.K. (2013). Traveling towards disease: transportation barriers to health care access. J. Community Health.

[bib82] Triana C.A., Sarmiento O.L., Bravo-Balado A., González S.A., Bolívar M.A., Lemoine P., Meisel J.D., Grijalba C., Katzmarzyk P.T. (2019). Active streets for children: the case of the Bogotá Ciclovía. PloS One.

[bib83] UN General Assembly (2015). Transforming our world : the 2030 agenda for sustainable development, UN general assembly.

[bib84] United Nations (2014). World Urbanization Prospects: the 2014 Revision, Highlights, Department of Economic and Social Affairs; Population Division.

[bib85] United Nations (2016). Mobilizing Sustainable Transport for Development: Analysis and Policy Recommendations from the United Nations Secretary-General's High-Level Advisory Group on Sustainable Transport.

[bib86] United Nations (2017). The new urban agenda. HABITAT III: United Nations Conference on Housing and Sustainable Urban Development.

[bib87] United Nations (2018). Revision of world urbanization prospects, population division of the united Nations department of economic and social affairs.

[bib88] van Sluijs E.M.F., McMinn A.M., Griffin S.J. (2007). Effectiveness of interventions to promote physical activity in children and adolescents: systematic review of controlled trials. BMJ.

[bib89] Venn D., Strazdins L. (2017). Your money or your time? How both types of scarcity matter to physical activity and healthy eating. Soc. Sci. Med..

[bib90] Vlahov D., Galea S., Freudenberg N. (2005). The urban health “advantage”. J. Urban Health : Bull. N. Y. Acad. Med..

[bib91] Weichenthal S., Hatzopoulou M., Brauer M. (2019). A picture tells a thousand…exposures: opportunities and challenges of deep learning image analyses in exposure science and environmental epidemiology.". Environ. Int..

[bib93] Yanez-Pagans P., Martinez D., Mitnik O.A., Scholl L., Vazquez A. (2018). Urban Transport Systems in Latin America and the Caribbean: Challenges and Lessons Learned.

[bib94] Yang L., Sahlqvist S., McMinn A., Griffin S.J., Ogilvie D. (2010). Interventions to promote cycling: systematic review. Br. Med. J..

[bib95] Yang Y., Auchincloss A.H., Rodriguez D.A., Brown D.G., Riolo R., Diez-Roux A.V. (2015). Modeling spatial segregation and travel cost influences on utilitarian walking: towards policy intervention." Computers. Environ. Urban Syst..

[bib96] Yang Y., Diez-Roux A. (2013). "Using an agent-based model to simulate children's active travel to school.". Int. J. Behav. Nutr. Phys. Activ..

[bib97] Yin L., Cheng Q., Wang Z., Shao Z. (2015). ‘Big data’ for pedestrian volume: exploring the use of Google Street View images for pedestrian counts. Appl. Geogr..

[bib98] Zellner M., Massey D., Shiftan Y., Levine J., Arquero M. (2016). Overcoming the last-mile problem with transportation and land-use improvements: an agent-based approach. Int. J. Trans..

[bib99] Zou M., Li M., Lin X., Xiong C., Mao C., Wan C., Zhang K., Yu J. (2016). An agent-based choice model for travel mode and departure time and its case study in Beijing.. Transport. Res. C Emerg. Technol..

[bib92] Wells, G.A., Shea, B., O'Connell, D., Peterson, J., Welch, V., Losos, M., Tugwell, P., 2013. The Newcastle-Ottawa Scale (NOS) for assessing the quality of nonrandomised studies in meta-analyses. http://www.ohri.ca/programs/clinical_epidemiology/nosgen.pdf (accessed 15 April 2019).

