# Supplementary material for: A systematic review of empirical and simulation studies evaluating the health impact of transportation interventions
Source: Environ Res. 2020 Jul;186:109519. doi: 10.1016/j.envres.2020.109519 (PMC7343239; doi:10.1016/j.envres.2020.109519)
Supplement: Multimedia component 1 [file mmc1.docx]

**Appendix 1: Comprehensive search strategy by database**

| **Medline (Ovid)**  1 *Disease/  2 *Health/  3 *Public Health/  4 *Urban Health/  5 *"Wounds and Injuries"/  6 *Mortality/  7 (health or disease* or behavio* or mortalit* or injur*).ti,ab.  8 1 or 2 or 3 or 4 or 5 or 6 or 7  9 ("Aerial lift*" or "aerial tram*" or "cable car*" or metrocable* or "gondola lift*" or "gondola car*" or   "cable propelled transit" or CPT).ti,ab.  10 ("Bus rapid transit" or BRT*).ti,ab.  11 (Ciclovia* or "mass event*" or "mega event*" or “open street*”).ti,ab.  12 *Bicycling/  13 ("Bike lane*" or "bike way*" or "bike path*" or bikeway* or cicloruta* or bicycl* or cycling).ti,ab.  14 12 or 13  15 9 or 10 or 11 or 14  16 *Non-Randomized Controlled Trials as Topic/  17 *Follow-Up Studies/  18 *Controlled Before-After Studies/  19 ("Systematic review*" or "quasi-experiment*" or "social experiment" or "natural experiment*" or   "difference in difference*" or pre-post or evaluation or "impact assessment*" or   "before and after").ti,ab.  20 ("Systematic review*").ti,ab.  21 (Simulation* or "systems model*" or "agent-based model*" or "multi-agent model*" or   "individual-based model*" or "system dynamics").ti,ab.  22 16 or 17 or 18 or 19 or 21  23 8 and 15 and 22  24 limit 23 to (humans and yr="2000-Current") |
| --- |
| **Scopus**  ( ( TITLE-ABS ( health  OR  disease*  OR  behavio*  OR  mortalit*  OR  injur* ) )  AND  ( ( TITLE-ABS ( "Aerial lift*"  OR  "aerial tram*"  OR  "cable car*"  OR  metrocable*  OR  "gondola lift*"  OR  "gondola car*"  OR  "cable propelled transit" ) )  OR  ( TITLE-ABS ( "Bus rapid transit"  OR  {BRT*} ) )  OR  ( TITLE-ABS ( ciclovia*  OR  "mass event*"  OR  "mega event*" OR “open street*” ) )  OR  ( TITLE-ABS ( {Bike lane*}  OR  {bike way*}  OR  {bike path*}  OR  bikeway*  OR  cicloruta*  OR  bicycl*  OR  bike ) ) ) )  AND  ( ( TITLE-ABS ( {Systematic review*}  OR  "quasi experiment*"  OR  {social experiment}  OR  {natural experiment*}  OR  "difference in difference*"  OR  pre-post  OR  evaluation  OR  {impact assessment*}  OR  {before and after} ) )  OR  ( TITLE-ABS ( simulation*  OR  {system* model*}  OR  {agent-based model*}  OR  {multi-agent model*}  OR  {individual-based model*}  OR  {system dynamics} ) ) )  AND  ( LIMIT-TO ( PUBYEAR ,  2017 )  OR  LIMIT-TO ( PUBYEAR ,  2016 )  OR  LIMIT-TO ( PUBYEAR ,  2015 )  OR  LIMIT-TO ( PUBYEAR ,  2014 )  OR  LIMIT-TO ( PUBYEAR ,  2013 )  OR  LIMIT-TO ( PUBYEAR ,  2012 )  OR  LIMIT-TO ( PUBYEAR ,  2011 )  OR  LIMIT-TO ( PUBYEAR ,  2010 )  OR  LIMIT-TO ( PUBYEAR ,  2009 )  OR  LIMIT-TO ( PUBYEAR ,  2008 )  OR  LIMIT-TO ( PUBYEAR ,  2007 )  OR  LIMIT-TO ( PUBYEAR ,  2006 )  OR  LIMIT-TO ( PUBYEAR ,  2005 )  OR  LIMIT-TO ( PUBYEAR ,  2004 )  OR  LIMIT-TO ( PUBYEAR ,  2003 )  OR  LIMIT-TO ( PUBYEAR ,  2002 )  OR  LIMIT-TO ( PUBYEAR ,  2001 )  OR  LIMIT-TO ( PUBYEAR ,  2000 ) )  AND  ( LIMIT-TO ( LANGUAGE ,  "English" )  OR  LIMIT-TO ( LANGUAGE ,  "Spanish" )  OR  LIMIT-TO ( LANGUAGE ,  "Portuguese" ) )  *NOTE: the keyword “cycling” was omitted because it created too much noise (i.e., it identified a lot of biochemistry papers).* |
| **TRID**  1 (health or disease* or behavio* or mortalit* or injur*).ti,ab.    2 "Aerial lift*" or "aerial tram*" or "cable car*" or metrocable* or "gondola lift*" or "gondola car*" or   "cable propelled transit" or CPT  3 "Bus rapid transit" or BRT*  4 Ciclovia* or "mass event*" or "mega event*" or "open street*"  5 "Bike lane*" or "bike way*" or "bike path*" or bikeway* or cicloruta* or bicycl* or cycling  6 2 or 3 or 4 or 5    7 1 and 6  *NOTE: Remaining keywords related to study design would not all load when entered into the database, so they were omitted. This search is therefore broader than the others.* |
| **LILACS**  ***Spanish***  1 (mh:(Enfermedad))  2 (mh:(Salud))  3 (mh:("Salud Pública"))  4 (mh:("Salud Urbana"))  5 (mh:("Heridas y Lesiones"))  6 (mh:(Mortalidad))  7 (ti:(salud or enfermed* or comportamiento* or mortalidad* or muert* or lesion*))  8 (ab:(salud or enfermed* or comportamiento* or mortalidad* or muert* or lesion*))  9 1 or 2 or 3 or 4 or 5 or 6 or 7 or 8    10 (ti:(teleférico* or telecabina* or "cable* aéreo*" or metrocable* or "transporte aéreo por cable"))  11 (ab:(teleférico* or telecabina* or "cable* aéreo*" or metrocable* or "transporte aéreo por cable"))  12 (ti:("bus rapid transit" or BRT* or "autobus* de tránsito rápido" or metrobus* or "transporte rápido en autobus*" or  "autobus* expreso*"))  13 (ab:("bus rapid transit" or BRT* or "autobus* de tránsito rápido" or metrobus* or "transporte rápido en autobus*" or  "autobus* expreso*"))  14 (ti:(ciclovía* or "calle* abierta*" or "evento* de masa*" or "mega evento*"))  15 (ab:(ciclovía* or "calle* abierta*" or "evento* de masa*" or "mega evento*"))  16 (mh:(ciclismo))  17 (ti:(ciclovía* or bicicarril* or bicisenda* or ciclorruta* or "vía* ciclista" or ciclopista* or bicicleta*))  18 (ab:(ciclovía* or bicicarril* or bicisenda* or ciclorruta* or "vía* ciclista" or ciclopista* or bicicleta*))  19 16 or 17 or 18  20 10 or 11 or 12 or 13 or 14 or 15 or 19    21 (mh:("Ensayos Clínicos Controlados como Asunto"))  22 (mh:("Estudios de Seguimiento"))  23 (mh:("Estudios Controlados Antes y Después"))  24 (ti:("revis* sistemática*" or "quasi-experiment*" or "quase-experiment*" or "experimento* socia*" or   "experimento* natura*" or "diferencia* en diferencia*" or "pre y pos" or evaluación or "evaluación de impacto" or  "antes y después"))  25 (ab:("revis* sistemática*" or "quasi-experiment*" or "quase-experiment*" or "experimento* socia*" or   "experimento* natura*" or "diferencia* en diferencia*" or "pre y pos" or evaluación or "evaluación de impacto" or  "antes y después"))  26 (ti:(simulacion* or "model* de sistema*" or "model* basad* en agentes" or "model* multiagentes" or   "model* multi-agentes" or "model* basad* en indivíduos" or "dinámica* de sistema*"))  27 (ab:(simulacion* or "model* de sistema*" or "model* basad* en agentes" or "model* multiagentes" or   "model* multi-agentes" or "model* basad* en indivíduos" or "dinámica* de sistema*"))  28 21 or 22 or 23 or 24 or 25 or 26 or 27  29 9 and 20 and 28  30 limit 29 to (humans and yr="2000-Current")  ***Portuguese***  1 (mh:(Doença))  2 (mh:(Saúde))  3 (mh:("Saúde Pública"))  4 (mh:("Saúde da População Urbana"))  5 (mh:("Ferimentos e Lesões"))  6 (mh:(Mortalidade))  7 (ti:(saúde or doen* or comportamento* or mort* or lesão or lesões or lesionad*))  8 (ab:(saúde or doença* or comportamento* or mort* or lesão or lesões or lesionad*))  9 1 or 2 or 3 or 4 or 5 or 6 or 7 or 8    10 (ti:(teleférico* or "transporte aéreo por cabo*"))  11 (ab:(teleférico* or "transporte aéreo por cabo*"))  12 (ti:("bus rapid transit" or BRT* or " transporte rápido por ônibus" or "veículo* leve* sobre pneu*"))  13 (ab:("bus rapid transit" or BRT* or " transporte rápido por ônibus" or "veículo* leve* sobre pneu*"))  14 (ti:(ciclovia* or ciclofaixa or "rua* aberta*" or "rua* de lazer" or "evento* de massa*" or "mega evento*" or  "megaevento"))  15 (ab:(ciclovia* or ciclofaixa or "rua* aberta*" or "rua* de lazer" or "evento* de massa*" or "mega evento*" or  "megaevento"))  16 (mh:(ciclismo))  17 (ti:(ciclovia* or ciclofaixa* or ciclorrota* or ciclopista* or bicicleta*))  18 (ab:(ciclovia* or ciclofaixa* or ciclorrota* or ciclopista* or bicicleta*))  19 16 or 17 or 18  20 10 or 11 or 12 or 13 or 14 or 15 or 19    21 (mh:("Ensaios Clínicos Controlados como Assunto"))  22 (mh:(Seguimentos))  23 (mh:("Estudos Controlados Antes e Depois"))  24 (ti:("revis* sistemática*" or "quasi-experiment*" or "quase-experiment*" or "experimento* socia*" or   "experimento* natura*" or "diferença* em diferença*" or "pré e pós" or avaliação or "avaliação de impacto" or   "antes e depois"))  25 (ab:("revis* sistemática*" or "quasi-experiment*" or "quase-experiment*" or "experimento* socia*" or   "experimento* natura*" or "diferença* em diferença*" or "pré e pós" or avaliação or "avaliação de impacto" or   "antes e depois"))  26 (ti:(simulaç* or "model* de sistema*" or "model* basead* em agentes" or "model* multiagentes" or   "model* multi-agentes" or "model* basead* em indivíduos" or "dinâmica* de sistema*"))  27 (ab:(simulaç* or "model* de sistema*" or "model* basead* em agentes" or "model* multiagentes" or   "model* multi-agentes" or "model* basead* em indivíduos" or "dinâmica* de sistema*"))  28 21 or 22 or 23 or 24 or 25 or 26 or 27    29 9 and 20 and 28  30 limit 29 to (humans and yr="2000-Current") |
