# Supplementary material for: A systematic review of empirical and simulation studies evaluating the health impact of transportation interventions
Source: Environ Res. 2020 Jul;186:109519. doi: 10.1016/j.envres.2020.109519 (PMC7343239; doi:10.1016/j.envres.2020.109519)
Supplement: Multimedia component 3 [file mmc3.docx]

**Appendix 3: Summary of outcomes assessed in included studies, by outcome type**

**TABLE 1: Summary of outcomes (n=162) assessed across all studies included in the review***

| **OUTCOME TYPE** | | **Outcomes** (N) | **Significant** (n) | **Significant** (%) | **Of the significant outcomes** | | |
| --- | --- | --- | --- | --- | --- | --- | --- |
|  |  |  |  |  | **Unexpected** (n) | **Expected** (n) | **Expected** (%) |
| Physiological |  | 2 | 2 | 100 | 0 | 2 | 100 |
| Anthropometric |  | 1 | 1 | 100 | 0 | 1 | 100 |
| Physical activity |  | 13 | 3 | 23 | 0 | 3 | 100 |
| Mode share |  | 36 | 20 | 56 | 1 | 19 | 95 |
| Active transport | trips | 17 | 8 | 47 | 2 | 6 | 75 |
|  | distance | 6 | 3 | 50 | 2 | 1 | 33 |
|  | distance share | 1 | 0 | 0 |  |  |  |
|  | duration | 41 | 20 | 49 | 5 | 15 | 75 |
|  | time share | 2 | 0 | 0 |  |  |  |
|  | frequency | 1 | 0 | 0 |  |  |  |
| Car travel | distance | 2 | 2 | 100 | 1 | 1 | 50 |
|  | duration | 2 | 1 | 50 | 1 | 0 | 0 |
| Injury |  | 37 | 6 | 16 | 4 | 2 | 33 |
| Homicide |  | 1 | 1 | 100 | 0 | 1 | 100 |
| ***TOTAL*** | | ***162*** | ***67*** |  | ***16*** | ***51*** |  |

** Does not include 21 outcomes reported by 5 studies because these outcomes were not tested for statistical significance*

**TABLE 2: Summary of outcomes (n=117) for studies assessing single component interventions***

| **OUTCOME TYPE** | | **Outcomes** (N) | **Significant** (n) | **Significant** (%) | **Of the significant outcomes** | | |
| --- | --- | --- | --- | --- | --- | --- | --- |
|  |  |  |  |  | **Unexpected** (n) | **Expected** (n) | **Expected** (%) |
| Physiological |  | 0 |  |  |  |  |  |
| Anthropometric |  | 0 |  |  |  |  |  |
| Physical activity |  | 7 | 0 | 0 |  |  |  |
| Mode share |  | 13 | 8 | 62 | 1 | 7 | 88 |
| Active transport | trips | 16 | 8 | 50 | 2 | 6 | 75 |
|  | distance | 6 | 3 | 50 | 2 | 1 | 33 |
|  | distance share | 1 | 0 | 0 | 0 | 0 | 0 |
|  | duration | 30 | 15 | 50 | 5 | 10 | 67 |
|  | time share | 2 | 0 | 0 |  |  |  |
|  | frequency | 1 | 0 | 0 |  |  |  |
| Car travel | distance | 2 | 2 | 100 | 1 | 1 | 50 |
|  | duration | 2 | 1 | 50 | 1 | 0 | 0 |
| Injury |  | 37 | 6 | 16 | 4 | 2 | 33 |
| Homicide |  | 0 |  |  |  |  |  |
| ***TOTAL*** | | ***117*** | ***43*** |  | ***16*** | ***27*** |  |

** Does not include 21 outcomes reported by 5 studies because these outcomes were not tested for statistical significance*

**TABLE 3: Summary of outcomes (n=45) for studies assessing multicomponent interventions**

| **OUTCOME TYPE** | | **Outcomes** (N) | **Significant** (n) | **Significant** (%) | **Of the significant outcomes** | | |
| --- | --- | --- | --- | --- | --- | --- | --- |
|  |  |  |  |  | **Unexpected** (n) | **Expected** (n) | **Expected** (%) |
| Physiological |  | 2 | 2 | 100 | 0 | 2 | 100 |
| Anthropometric |  | 1 | 1 | 100 | 0 | 1 | 100 |
| Physical activity |  | 6 | 3 | 50 | 0 | 3 | 100 |
| Mode share |  | 23 | 12 | 52 | 0 | 12 | 100 |
| Active transport | trips | 1 | 0 | 0 |  |  |  |
|  | distance | 0 |  |  |  |  |  |
|  | distance share | 0 |  |  |  |  |  |
|  | duration | 11 | 5 | 45 | 0 | 5 | 100 |
|  | time share | 0 |  |  |  |  |  |
|  | frequency | 0 |  |  |  |  |  |
| Car travel | distance | 0 |  |  |  |  |  |
|  | duration | 0 |  |  |  |  |  |
| Injury |  | 0 |  |  |  |  |  |
| Homicide |  | 1 | 1 | 100 | 0 | 1 | 100 |
| ***TOTAL*** | | ***45*** | ***24*** |  | ***0*** | ***24*** |  |
