# Supplementary material for: A systematic review of empirical and simulation studies evaluating the health impact of transportation interventions
Source: Environ Res. 2020 Jul;186:109519. doi: 10.1016/j.envres.2020.109519 (PMC7343239; doi:10.1016/j.envres.2020.109519)
Supplement: Multimedia component 4 [file mmc4.docx]

**Table 5: Quality assessment of evaluation studies**

|  |  | **SAMPLING** | | **COMPARABILITY** | | **OUTCOME** | | | |
| --- | --- | --- | --- | --- | --- | --- | --- | --- | --- |
| **ID** | **STUDY** | **Sampling strategy** | **Representativeness of recruited sample** | **Control/ reference group?** | **Intervention & control/reference groups similar at baseline?** | **Outcome assessment** | **Timing (duration) of intervention assessment** | **Adequacy of follow-up** | **Attrition*** |
| 1 | Bhatia et al.  (2016) | Attempt made to sample entire study area | Somewhat representative | No | NA | Objectively measured & self-reported | T1: 23-month period ending 1 month before intervention T2: 23-month period immediately after intervention | NA |  |
| 2 | Boarnet et al. (2005) | Observational counts | Not very representative | No | NA | Objectively measured | Unclear | NA |  |
| 3 | Brown et al.  (2016) | Attempt made to sample entire study area | Unclear | Yes | Unclear | Objectively measured | T1: unclear ~ 12 months before intervention T2: 6-month period starting 1 month after intervention | Some loss to follow-up | 41% |
| 4 | Brown et al. (2016b) | No description of sampling strategy | Unclear | Yes | Unclear | Objectively measured & self-reported | T1: unclear ~ 12 months before intervention T2: 6-month period starting 1 month after intervention | Some loss to follow-up | 41% |
| 5 | Burbidge et al. (2009) | Non-random sampling/ stratification | Somewhat representative | No | NA | Self-reported | T1: 7 months before intervention T2: 1 month after intervention T3: 5 months after intervention | Some loss to follow-up | 87.7% |
| 6 | Cerdà et al.  (2012) | Random sampling | Somewhat representative | Yes | Yes, quantitative characterization of samples provided | Objectively measured | T1: ~ 12 months before intervention T2: 4 years after intervention | Some loss to follow-up | 22% |
| 7 | Chang et al.  (2017) | Random sampling | Unclear | No | NA | Self-reported | T1: 24 months before intervention T2: 12 months after intervention | NA |  |
| 8 | Chen et al.  (2012) | Attempt made to sample entire study area | Somewhat representative | Yes | Yes, quantitative characterization of samples provided | Objectively measured & self-reported | T1: 5-year period immediately before intervention T2: 2-year period immediately after intervention | NA |  |
| 9 | Cook et al.  (2016) | Observational counts | Unclear | No | NA | Objectively measured & self-reported | T1: 8 months before intervention T2: 3 months after intervention | NA |  |
| 10 | Dill et al.  (2014) | Attempt made to sample entire study area | Unclear | Yes | Yes, quantitative characterization of samples provided | Objectively measured | Timing unclear as intervention dates are not reported.  T1: Pre-intervention assessments: mid Jul 2010 to Sept 2011 T2: Post-intervention assessments: Aug 2012 to Aug 2013 | Some loss to follow-up | 28% |
| 11 | Evenson et al. (2005) | Random sampling | Unclear | No | NA | Self-reported | T1: 1 year & 5 months before intervention T2: 2 months after intervention | Some loss to follow-up | 47% |
| 12 | Ferenchak et al. (2016) | Attempt made to sample entire study area | Somewhat representative | No | NA | Objectively measured & self-reported | T1: 2-year period starting 1-3 years before intervention T2: 2-year period starting 1-2 years after intervention | NA |  |
| 13 | Goodman et al. (2013) | Attempt made to sample entire study area | Truly representative | Yes | Yes, general statements made | Self-reported | T1: 24 years before intervention. T2: 14 years before intervention. T3: 4 years before intervention. T4: unclear - immediately after intervention | NA |  |
| 14 | Goodman et al. (2013b) | Attempt made to sample entire study area | Unclear | No | NA | Self-reported | T1: 3 months before intervention T2: 1 year after intervention T3: 2 years after intervention | Some loss to follow-up | 57% |
| 15 | Goodman et al. (2014) | Attempt made to sample entire study area | Unclear | No | NA | Self-reported | T1: 3 months before intervention T2: 1 year after intervention T3: 2 years after intervention | Some loss to follow-up | 58% |
| 16 | Greaves et al. (2015) | Purposive sampling | Unclear | Yes | Unclear | Objectively measured & self-reported | T1: 3-month period starting 3 months before intervention T2: 4 months after intervention | Some loss to follow-up | 49% |
| 17 | Heesch et al. (2016) | Purposive sampling | Unclear | Yes | Yes, quantitative characterization of samples provided | Objectively measured & self-reported | T1: 11 months before intervention T2: 3-month period starting 3 months after intervention | NA |  |
| 18 | Heinen et al.  (2015) | Purposive sampling | Unclear | No | NA | Self-reported | T1: 2 years before intervention T2: 1 year after intervention | Some loss to follow-up | 57% |
| 19 | Heinen et al.  (2016) | Purposive sampling | Unclear | No | NA | Self-reported | T1: 2 years before intervention T2: 1 year after intervention | Some loss to follow-up | 57% |
| 20 | Jensen  (2008) | No description of sampling strategy | Unclear | Yes | Unclear | Objectively measured | T1: 5-year period starting 8-12 years before intervention T2: unclear | NA |  |
| 21 | Langdon  (2015) | Non-random sampling/ stratification | Unclear | No | NA | Objectively measured & self-reported | T1: 2 to 47 months before intervention T2: 26 to 58 months after intervention Timing of traffic counts unclear | NA |  |
| 22 | Panter et al.  (2015) | Attempt made to sample entire study area | Unclear | Yes | Unclear | Self-reported | T1: 3 to 17 months before intervention T2: 7 to 27 months after intervention | Some loss to follow-up | 57% |
| 23 | Panter et al.  (2016) | Non-random sampling/ stratification | Unclear | No | NA | Self-reported | T1: 5-month period ending 22 months before intervention T2: 5-month period starting 9 after intervention | Some loss to follow-up | 59% |
| 24 | Panter et al.  (2017) | Attempt made to sample entire study area | Unclear | Yes | Unclear | Self-reported | T1: 3 to 17 months before intervention T2: 15 months after the intervention T3: 7 to 27 months after intervention | Some loss to follow-up | 64% |
| 25 | Parker et al.  (2011) | Non-random sampling/ stratification | Unclear | No | NA | Objectively measured | T1: 6 months before intervention  T2: 6 months after intervention | NA |  |
| 26 | Parker et al.  (2013) | Non-random sampling/ stratification | Somewhat representative | Yes | Unclear | Objectively measured | T1: 9 months before intervention  T2: 3 months after intervention | NA |  |
| 27 | Pazin et al.  (2016) | Random sampling | Somewhat representative | Yes | Yes, quantitative characterization of samples provided | Self-reported | T1: 4-month period starting 16 months before intervention T2: 9-month period starting 20 months after intervention | Some loss to follow-up | 30.3% |
| 28 | Rissel et al.  (2015) | Non-random sampling/ stratification | Unclear | Yes | Yes, general statements made | Objectively measured & self-reported | T1: 8 months before intervention T2: 4 months after intervention | Some loss to follow-up | 40% |
| 29 | Song et al.  (2017) | Attempt made to sample entire study area | Unclear | Yes | Unclear | Self-reported | T1: 3 to 17 months before intervention T2: 15 months after intervention T3: 7 to 27 months after intervention | Some loss to follow-up | 58% |

NA: Not applicable; IA: immediately after; IB: immediately before; T1: time 1; T2: time 2; T3: time 3; T4: time 4

*Where >1 follow-up wave, attrition is reported for the last wave.
