# Supplementary material for: A systematic review of empirical and simulation studies evaluating the health impact of transportation interventions
Source: Environ Res. 2020 Jul;186:109519. doi: 10.1016/j.envres.2020.109519 (PMC7343239; doi:10.1016/j.envres.2020.109519)
Supplement: Multimedia component 5 [file mmc5.docx]

**Table 6: Quality appraisal of included system-based simulation studies**

| **ID** | **Author & year** | **Empirical sources used to inform parameters** | **Equations and justification provided** | **Justification for assumptions provided** | **Calibration conducted** | **Validation conducted** | **Sensitivity or uncertainty analysis** |
| --- | --- | --- | --- | --- | --- | --- | --- |
| 30 | Lemoine et al. (2016) | Yes, for all parameters | Yes, justification for some | Yes, one or few mentioned in passing | Yes, best-fit calibration | Yes, qualitative comparison between output and data/empirical patterns | Only sensitivity |
| 31 | Macmillan et al. (2014) | Yes, for all parameters | Yes, justification for some | Yes, all or most assumptions made explicit | Yes, multiple methods | Yes, qualitative and quantitative comparison between output and data/empirical patterns | Both |
| 32 | McDonnell & Zellner (2011) | Yes, for some parameters | No | Yes, all or most assumptions made explicit | No | Yes, qualitative and quantitative comparison between output and data/empirical patterns | Only sensitivity |
| 33 | Okushima & Akiyama (2011) | Yes, for some parameters | Yes, justification for all | Yes, all or most assumptions made explicit | No | No | Only sensitivity |
| 34 | Okushima (2015) | Yes, for all parameters | Yes, justification for all | Yes, all or most assumptions made explicit | Not required (data for all parameters) | No | No |
| 35 | Okushima (2016) | Yes, for all parameters | Yes, justification for all | Yes, all or most assumptions made explicit | Not required (data for all parameters) | No | No |
| 36 | Yang & Diez-Roux (2013) | Yes, for some parameters | Yes, justification for all | Yes, all or most assumptions made explicit | Yes, categorical calibration | No | No |
| 37 | Yang et al. (2015) | Yes, for all parameters | Yes, justification for some | Yes, all or most assumptions made explicit | Not required (data for all parameters) | Yes, qualitative comparison between output and data/empirical patterns | No |
| 38 | Zellner et al. (2016) | Yes, for all parameters | Yes, justification for some | Yes, all or most assumptions made explicit | Yes, categorical calibration | No | Only sensitivity |
| 39 | Zou et al. (2016) | Yes, for some parameters | Yes, justification for all | Yes, all or most assumptions made explicit | Yes, categorical calibration | No | No |
